# Development of resistance to FGFR inhibition in urothelial carcinoma via multiple pathways *in vitro*

**DOI:** 10.1002/path.6034

**Published:** 2022-12-13

**Authors:** Geoffrey A Pettitt, Carolyn D Hurst, Zubeda Khan, Helen R McPherson, Matthew C Dunning, Olivia Alder, Fiona M Platt, Emma VI Black, Julie E Burns, Margaret A Knowles

**Affiliations:** ^1^ Division of Molecular Medicine, Leeds Institute of Medical Research at St James's St James's University Hospital Leeds UK

**Keywords:** bladder cancer, FGFR3 inhibition, resistance mechanisms, transcriptome, HRAS mutation, IGF1R, YAP/TAZ, drug tolerance

## Abstract

Alterations of fibroblast growth factor receptors (FGFRs) are common in bladder and other cancers and result in disrupted signalling via several pathways. Therapeutics that target FGFRs have now entered the clinic, but, in common with many cancer therapies, resistance develops in most cases. To model this, we derived resistant sublines of two FGFR‐driven bladder cancer cell lines by long‐term culture with the FGFR inhibitor PD173074 and explored mechanisms using expression profiling and whole‐exome sequencing. We identified several resistance‐associated molecular profiles. These included *HRAS* mutation in one case and reversible mechanisms resembling a drug‐tolerant persister phenotype in others. Upregulated IGF1R expression in one resistant derivative was associated with sensitivity to linsitinib and a profile with upregulation of a YAP/TAZ signature to sensitivity to the YAP inhibitor CA3 in another. However, upregulation of other potential therapeutic targets was not indicative of sensitivity. Overall, the heterogeneity in resistance mechanisms and commonality of the persister state present a considerable challenge for personalised therapy. Nevertheless, the reversibility of resistance may indicate a benefit from treatment interruptions or retreatment following disease relapse in some patients. © 2022 The Authors. *The Journal of Pathology* published by John Wiley & Sons Ltd on behalf of The Pathological Society of Great Britain and Ireland.

## Introduction

Aberrant fibroblast growth factor receptor (FGFR) signalling occurs in many cancers [1, 2], most commonly through amplification or mutation of FGFRs. These mechanisms of activation are somewhat tissue specific. In lung [3, 4] and breast cancers [5, 6] *FGFR1* is amplified, in gastric cancer *FGFR2* is amplified [7]. Activating point mutations are found in *FGFR2* in endometrial cancer [8] and in *FGFR3* in bladder cancer [9]. Receptor activation can also occur following DNA rearrangements; this includes juxtaposition of *FGFR3* with the IGH regulatory region in multiple myeloma due to a t(4:14) translocation [10], generation of FGFR fusion proteins including FGFR3–TACC3 in glioblastoma [11], bladder [12] and other tumour types, and FGFR2 fusions with several partners in cholangiocarcinoma [13] (reviewed in [2]).

The high frequency of alterations, the broad range of cancers affected and preclinical evidence that they are potent drivers of the malignant phenotype have led to the development of a range of inhibitors and multiple clinical trials [14]. Such inhibitors may have an important therapeutic role in muscle‐invasive bladder cancer (MIBC), where activation of FGFR3 signalling by point mutation, gene fusion and/or upregulated expression is found in ~50% of cases [15, 16]. Preclinical studies show that bladder tumour cell lines with *FGFR3* point mutations or gene fusions are sensitive to FGFR inhibition [17, 18, 19], and good responses of bladder cancer patients in some phase 1 trials [20] have led to trials of FGFR inhibitors in patients with FGFR‐altered urothelial carcinoma, with promising results [21, 22]. US Food and Drug Administration (FDA) breakthrough therapy designation has been given for erdafitinib, a selective small‐molecule FGFR inhibitor, for treatment of locally advanced or metastatic urothelial cancer containing *FGFR3* or *FGFR2* alterations that has advanced following platinum‐containing chemotherapy. However, as with other targeted therapies, resistance to FGFR‐targeted agents is a problem. Indeed, although initial response rates have been encouraging, response rates remain below 50% and response duration has been short‐lived [21, 22].

Since tumours contain multiple molecular alterations, the response to targeting specific signalling proteins or pathways in tumour cells may be complicated by the reactivation of downstream elements of the targeted pathway and/or alterations in alternative pathways. Studies using cultured cells have examined mechanisms of short‐term cell survival and acquired resistance to FGFR‐targeted agents. Expression of a cDNA library encoding secreted proteins demonstrated that the EGFR family ligands NRG1, NRG2 and TGF‐alpha and the MET ligand HGF could rescue the FGFR3–TACC3 fusion‐driven bladder tumour cell line RT112 from FGFR inhibition [23]. Similar results were reported in rescue screens with exogenous growth factors in RT112 and a second FGFR3–TACC3‐containing bladder cell line, RT4 [24], and an siRNA screen implicated EGFR activation in limiting short‐term sensitivity of these lines and MGH‐U3, which has point‐mutated *FGFR3*, to the small‐molecule inhibitor PD173074 [25].

Other studies examined acquired resistance following long‐term culture in the presence of FGFR inhibitors. In the FGFR3‐mutant myeloma cell line KMS‐11, this produced a stable resistant derivative with a mutation (V555M) in the gatekeeper residue of FGFR3 that is predicted to limit drug access to the ATP binding site [26]. Resistance mediated via activation of ERBB2 and ERBB3 [27] or by enrichment of expression of RAS and MAPK pathway genes [28] has been reported following long‐term culture of RT112 with the FGFR1‐3 inhibitor infigratinib. However, in another study, resistance to infigratinib did not involve changes in EGFR, ERBB2 or MET signalling [29], indicating that different mechanisms of resistance may arise in the same cell line.

To study diversity in resistance mechanisms and identify potential targets for combination therapy, we derived resistant variants of FGFR3–TACC3 fusion‐driven urothelial tumour cell lines. These were examined by whole‐exome sequencing, copy number and transcriptome analysis to uncover genetic and gene expression changes associated with resistance. Resistance involved both reversible and stable mutational mechanisms. Our data implicate multiple pathways to resistance and show that identification of changes in expression of other targets does not invariably predict sensitivity. This has important implications for treatment regimens with these inhibitors.

## Materials and methods

### Cell culture and reagents

RT112M and RT4 were cultured in RPMI‐1640 and McCoy's 5A respectively (Sigma‐Aldrich, St. Louis, MO, USA) supplemented with 10% FCS. Resistant derivatives were derived and maintained in medium containing 1 μm (R1, R3) or 2 μm (R2) PD173074. Cell viability assays used CellTiter Blue (Promega, Southampton, UK). Further details are given in Supplementary materials and methods.

Small‐molecule inhibitors used in this study were PD173074 (Sigma Aldrich), erlotinib (NSC 718781; Cayman Chemical, Ann Arbor, MI, USA), sapitinib (AZD8931; ApexBio Technology, Houston, TX, USA), linsitinib (OSI‐906; BioVision, Waltham, MA, USA), capmatinib (INC280; Adooq Bioscience, Irvine, CA, USA and Cayman Chemical), erdafitinib (JNJ‐42756493; Cayman Chemical), infigratinib (BGJ398; Selleck Biochemicals, Houston, TX, USA), GSK‐J4 HCl (Apex Biotechnology, Chennai, Tamil Nadu, India) and CA3 (CIL56; Cayman Chemical). All small‐molecule inhibitors were dissolved in dimethyl sulphoxide (DMSO) and stored at −20 or −80 °C.

Primary antibodies used for immunoblotting were β‐actin (1:3000, sc‐81178; Santa Cruz Biotechnology, Dallas, Texas, USA.), AKT (1:1000, #4691; Cell Signalling Technology, Danvers, MA, USA), E‐cadherin (1:500, ab1416; Abcam, Cambridge, UK), EGFR (1:1000, sc‐03; Santa Cruz Biotechnology), ERBB2 (1:1000, ab2428; Abcam), ERBB3 (1:1000, sc‐285; Santa Cruz Biotechnology), ERK (1:1000, sc‐94; Santa Cruz Biotechnology), FGFR3 (1:1000, sc‐13121; Santa Cruz Biotechnology), N‐cadherin (1:500, sc‐59987; Santa Cruz Biotechnology), MET (1:1000, #4560; Cell Signalling Technology), phospho‐Akt (Ser473: 1:1000, #4060; Cell Signaling Technology), phospho‐EGFR (Tyr1068: 1:1000, #3777, Cell Signaling Technology), phospho‐ERBB2 (Tyr1221/1222: 1:1000, #2243; Cell Signaling Technology), phospho‐ERBB3 (Tyr1289: 1:1000, #4791; Cell Signaling Technology), phospho‐ERK (Tyr204: 1:1000, sc‐7383; Santa Cruz Biotechnology), phospho‐MET (Tyr1234/1235: 1:1000, #3077; Cell Signaling Technology), YAP/TAZ (1:1000, #8418; Cell Signaling Technology), IGF‐1R beta (1:1000, #9750; Cell Signaling Technology), phospho‐IGF‐1R beta/phospho‐InsR beta (1:1000 #3024; Cell Signaling Technology) and vimentin (1:500, sc‐5565; Santa Cruz Biotechnology).

### DNA and RNA extraction and molecular analysis

Genomic DNA was isolated using a Gentra Puregene Kit (Qiagen, Hilden, Germany). Whole‐exome sequencing was carried out using the SureSelect Human All Exon V6 Kit. Total RNA was isolated using an RNeasy Mini Kit (Qiagen) and analysed using Affymetrix Human Transcriptome 2.0 microarrays (Affymetrix, Santa Clara, CA, USA). Copy number alterations were examined by low pass genomic sequencing. Further details are given in Supplementary materials and methods.

### Transcriptome analysis

The R2 genomics analysis and visualization platform (http://r2.amc.nl) was used for data visualisation, data mining and analysis of transcriptome data. The statistical test LIMMA with FDR 0.01 was applied to identify genes differentially expressed between groups.

Gene Ontology (GO) (biological processes) was performed using the Database for Annotation and Integrated Discovery (DAVID) version 6.8. Gene Set Enrichment Analysis (GSEA) version 3.0 was carried out using all genes run against gene sets in the Hallmarks database (version 7.1). Details are given in Supplementary materials and methods.

### Statistics

Statistical analysis was carried out using GraphPad Prism 8.2 or 9.3.1 for Mac (GraphPad, San Diego, CA, USA). Group comparisons of single gene expression levels and gene signatures used one‐way ANOVA with Tukey's multiple comparison test or Student's *t*‐tests. Comparisons between drug treatments used non‐linear regression curve fit. IC_50_ values were calculated with 95% confidence limits and compared using unpaired two‐tailed *t*‐tests. A significance level of 0.05 was used.

## Results

### Derivation of resistant lines

**Figure 1:**
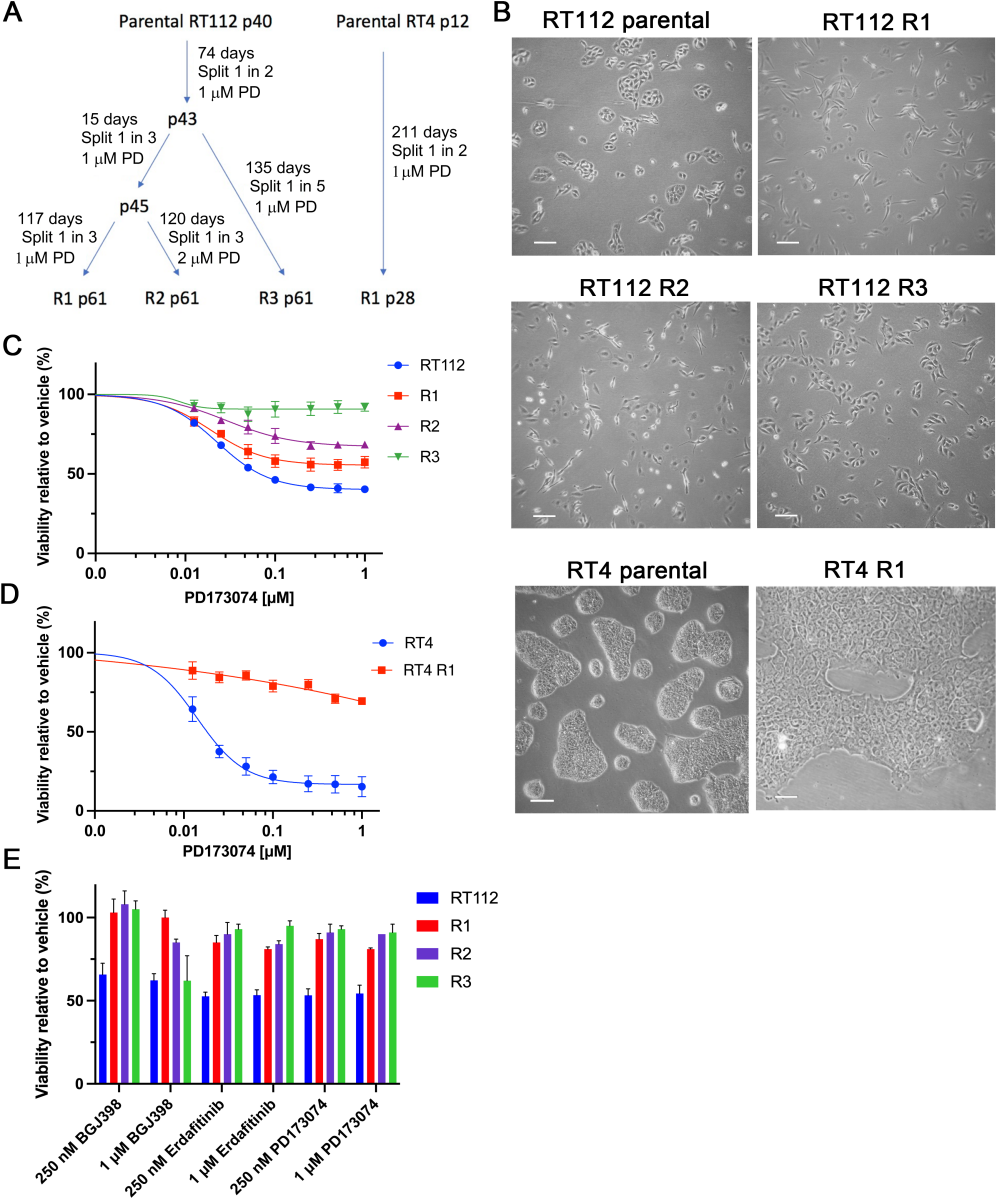
Derivation of PD173074 (PD) RT112 and RT4 resistant derivatives. (A) Derivation of RT112 and RT4 resistant derivatives from their parental lines. Passage number is denoted by a ‘p’ prior to the passage number. (B) Morphology of parental RT112 and RT4 cultured without PD and resistant derivatives cultured in PD. Scale bar, 100 μm. (C) Viability of RT112 parental and resistant derivatives in 0–1 μm PD. (D) Viability of RT4 parental and RT4 R1 in 0–1 μm PD. (E) Viability of RT112 and resistant derivatives in other FGFR inhibitors (mean + SEM of two (R1, R2) or three (RT112, R3) experiments). (C–E) Viability of cells was assayed following 120 h treatment with drug and normalised to vehicle control. Error bars show SEM of at least two assays. Sigmoidal dose response curves were plotted using GraphPad Prism.

In preclinical studies, RT112 and RT4 cell lines, which express both wild type and FGFR3–TACC3 fusion proteins [12], have shown greater sensitivity to FGFR inhibitors than other urothelial cell lines with FGFR3 alterations [17] and were selected for study. Derivatives resistant to the pan‐FGFR inhibitor PD173074 were selected by treatment at ~40% confluence with 1 μm PD173074 followed by long‐term culture (20 passages when 90% confluent, up to 210 days) in 1 μm or 2 μm PD173074 (Figure 1A). Chronic treatment at constant high dose rather than incrementally increased dose was chosen to mimic the situation where tumours are exposed immediately to high doses during *in vivo* treatment. Much cell death occurred during initial treatment of both cell lines, and cells proliferated at a very slow rate. Three RT112 resistant lines (RT112 R1, R2 and R3) and one RT4 resistant derivative (RT4 R1) were examined. With continued treatment, proliferation gradually increased, and during this time RT112 cells acquired a mesenchymal morphology, in contrast to the epithelial morphology of parental cells. Saturation density was lower in the resistant derivatives. Mesenchymal morphology was maintained by derivatives RT112 R1 and R2, whereas RT112 R3 precursor cells grew faster and regained epithelial morphology (Figure 1B). RT4 cells grow as a tall palisade of tightly clustered epithelial cells. Although the drug‐treated RT4 cells remained as clusters, cells were flatter and had more peripheral cytoplasmic extensions, though mesenchymal morphology was not acquired (Figure 1B).

When each line had resumed stable proliferation, viability assays were conducted (Figure 1C,D). PD173074‐resistant RT112 R1, R2 and R3 were cross‐resistant to clinically relevant FGFR inhibitors erdafitinib and infigratinib, although R3 was resistant only to the lower dose of infigratinib tested (250 nm) and sensitive to 1 μm (Figure 1E).

### Loss of FGFR3 expression and acquisition of mesenchymal markers in RT112 resistant lines

**Figure 2:**
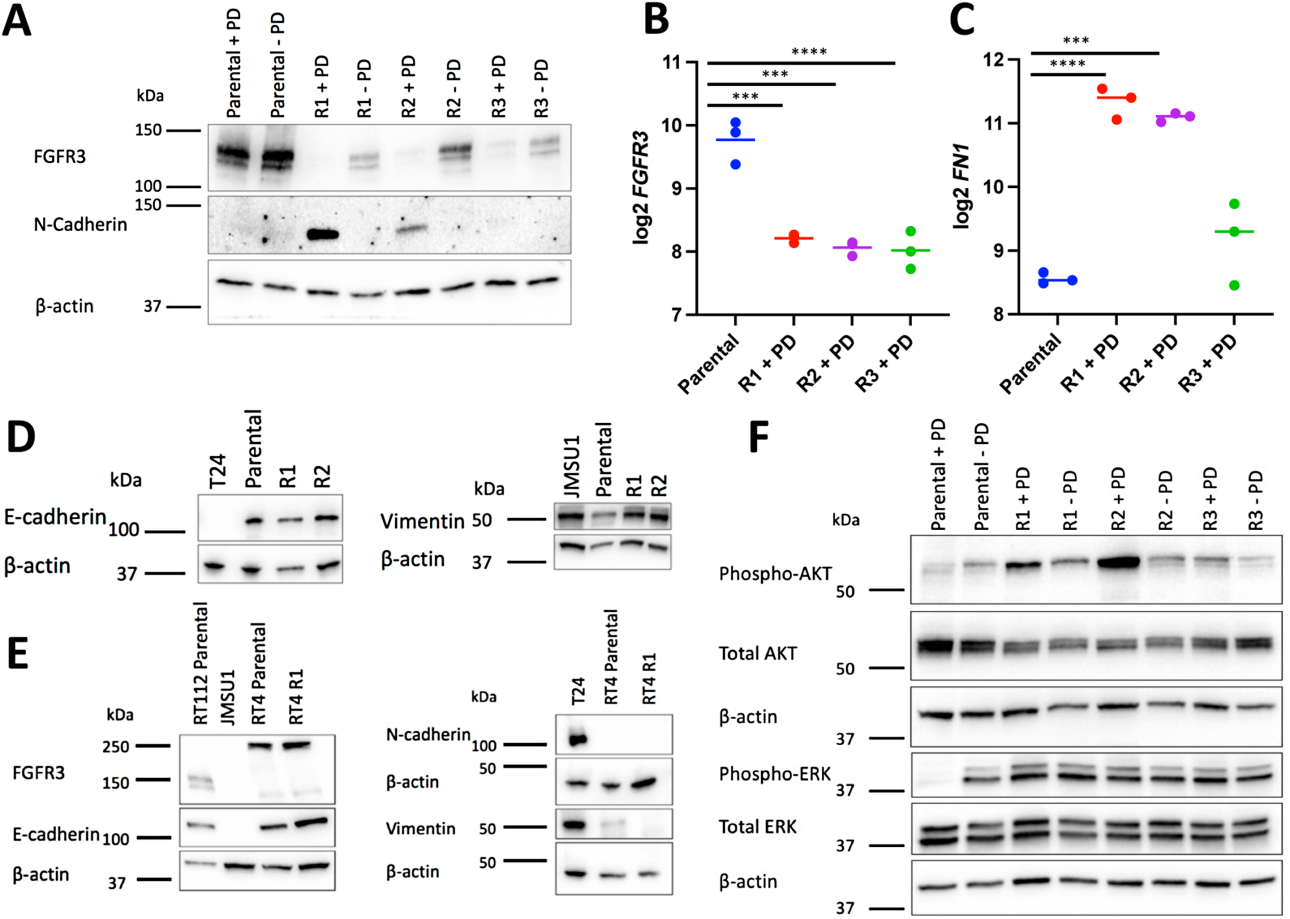
Protein expression and downstream signalling changes in RT112 and RT4 FGFR inhibitor‐resistant derivatives. (A) Immunoblot showing FGFR3 and N‐cadherin expression in RT112 and resistant derivatives. Parental RT112 + PD were cultured in 1 μm PD173074 for 24 h, R1, R2, R3 – PD were cultured without drug for four passages. (B) mRNA expression of *FGFR3* in RT112 parental cells and resistant derivatives. (C) mRNA expression of fibronectin 1 (*FN1*) in RT112 parental cells and resistant derivatives. (D) Immunoblots showing E‐cadherin and vimentin expression in RT112 parental cells and R1 and R2 resistant derivatives. T24 and JMSU1 were used as E‐cadherin‐negative and vimentin‐positive controls respectively. (E) Immunoblot showing FGFR3 and N‐cadherin expression in RT4. JMSU1 and T24 were used as FGFR3‐negative and N‐cadherin‐positive controls respectively. (F) Immunoblot showing phospho‐AKT, AKT, phospho‐ERK and ERK expression in parental RT112 and resistant derivatives. Parental RT112 + PD were cultured in 1 μm PD173074 for 24 h, R1, R2, R3 – PD were cultured without drug for four passages. (A), (D), (E) and (F). Immunoblots were conducted at least twice and representative examples shown. β‐actin was used as a loading control. (B, C) One‐way ANOVA with Tukey's multiple comparison test. **** *p* < 0.0001, ***p < 0.001.

FGFR3 expression is associated with an epithelial phenotype in bladder cancer cell lines [30]. In parental RT112, FGFR3 protein and mRNA expression was high but was reduced to strikingly low levels in their resistant derivatives (Figure 2A,B), implying that resistance to FGFR inhibition was not due to a mechanism that involved FGFR3 itself, such as a gatekeeper residue mutation. There was no change in the expression of FGFR1, FGFR2 or their isoforms in RT112 R1 or R2, suggesting that isoform switching was not a factor (data not shown). Because RT112 R1 and R2 had acquired mesenchymal morphology and drug‐resistant variants of RT112 have been reported to express mesenchymal markers [27], we sought evidence for an epithelial to mesenchymal transition (EMT). A significant increase in the expression of N‐cadherin was measured in RT112 R1 and R2 but not in R3 (Figure 2A). Similarly, expression of fibronectin (*FN1*) and slug (*SNAI2*) mRNA was increased in RT112 R1, R2 but not R3 (Figure 2C and supplementary material Figure S1A); a slight increase in levels of vimentin was apparent, but E‐cadherin levels were not significantly reduced (Figure 2D).

In contrast, drug‐resistant RT4 cells retained high levels of FGFR3 expression and showed no changes in E‐cadherin, N‐cadherin or vimentin expression (Figure 2E), although fibronectin (*FN1*) mRNA was increased (supplementary material Figure S1B). These results suggest that, in line with the observed morphological changes, R1 and R2 drug‐resistant derivatives of RT112 had undergone a partial EMT, and that this was less prominent in RT112 R3 and in RT4.

### Reversibility of drug resistance

**Figure 3:**
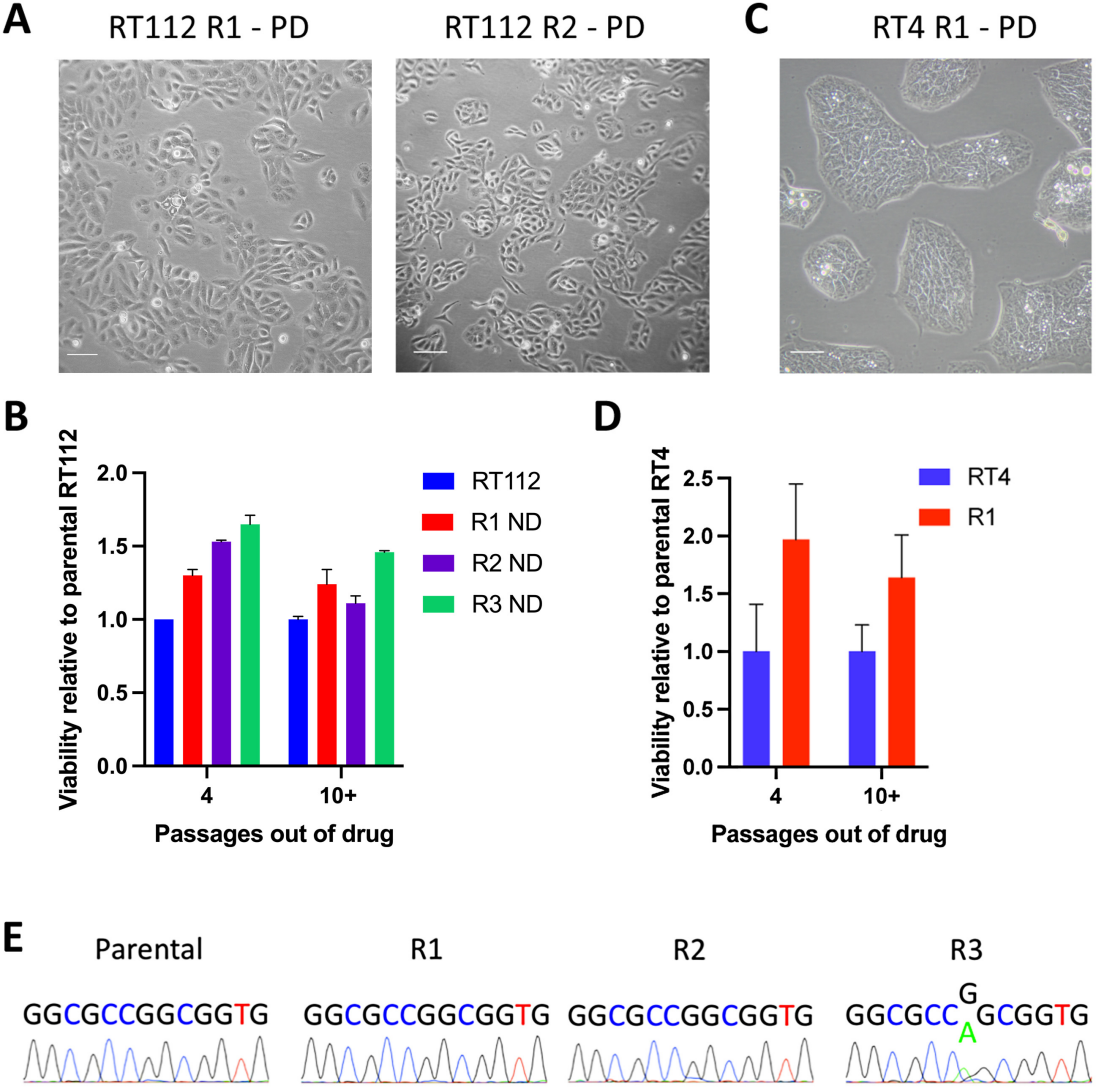
Reversibility of resistant phenotype. (A) Morphology of RT112 R1 and R2 cultured without PD173074 for four passages. Scale bar, 100 μm. (B) Viability relative to parental RT112 after culture without PD173074 for four or 10+ passages. ND, no drug. (C) Morphology of RT4 cultured without PD173074 for four passages. Scale bar, 100 μm. (D) Viability relative to parental RT4 of resistant derivative after culture without PD173074 for four or 10+ passages. (E) The mutation *HRAS* (G12S) is present in RT112 R3 but not in parental RT112 or other resistant derivatives. (B and D) Cell viability was assayed following 120 h treatment with drug and normalised to vehicle control. Error bars show SEM of at least two assays.

Resistant derivatives were cultured without drug for four passages (3–5 weeks). Proliferation of RT112 R1 and R2 increased, and cells regained epithelial morphology (Figure 3A). Levels of FGFR3 protein increased in all three resistant derivatives of RT112 but were not restored to levels found in parental cells (Figure 2A). After four passages in the absence of drug, cells were challenged with PD173074. RT112 R1 cells showed a partial reversion to sensitivity, whereas R2 and R3 retained a high degree of resistance. Sensitivity of RT112 R2 increased after more prolonged culture out of drug, but R3 resistance remained high (Figure 3B). RT4 R1 cells showed a minor change in morphology and reversion of sensitivity almost to the levels measured in parental cells after four passages out of drug (Figure 3C,D).

We examined levels of pERK and pAKT, both of which are active downstream of FGFR3 activation in RT112 [29] (Figure 2F). ERK phosphorylation was reduced by acute treatment with PD173074 (24 h) but was phosphorylated to a similar level in parental RT112 and all RT112 resistant derivatives, both in and out of drug. Phosphorylation of AKT Ser473 was reduced by acute treatment with drug but increased in R1 and R2 in the presence of drug. Levels of both pERK and pAKT in RT112 R3 were similar to those in untreated parental cells.

The reversibility of the resistant phenotype in RT112 R1 and R2 and RT4 R1 and the differential levels of AKT phosphorylation observed in the presence and absence of drug suggested that the underlying resistance mechanism(s) in these cells were not the result of a heritable genetic alteration. In contrast, the maintenance of resistance by RT112 R3 in the absence of drug and the lack of change in ERK and AKT phosphorylation levels indicated that a heritable mechanism might be responsible.

### Resistance in RT112 R3 is conferred by *HRAS* mutation

To determine whether the non‐reverting resistant phenotype of RT112 R3 was caused by genetic alteration, whole‐exome sequencing was carried out on R3 and parental RT112. Mutations present in the resistant derivative but not the parental line are listed in the supplementary material, Table S2. Notably, a known activating mutation in *HRAS* (G12S) was present in 131 of 179 (73%) reads in R3 and absent in parental cells. This mutation in R3 and its absence in R1 and R2 was confirmed by Sanger sequencing (Figure 3E). Copy number analysis using low‐pass whole‐genome sequencing and an unmatched normal reference DNA sample showed that parental RT112 and R3 exhibited loss of a region on chromosome 11 (11:0–8,295,312 Mb; hg38) that includes *HRAS* (supplementary material Figure S2 and Table S3). Because RT112 is diploid, this indicates that these lines have only one copy of *HRAS*. Consistent with this, SNaPshot analysis of 36 single‐cell clones of RT112 R3 identified 21 with *HRAS* G12S only, 14 with wild type only and one that appeared heterozygous. Further, single‐cell cloning of this apparently heterozygous sample confirmed that it was not of single‐cell origin since only mutant or wild type clones were obtained. This indicates that RT112 R3 is a mixed population of cells, each with a single wild type or mutant *HRAS* allele. The proportion of mutant *HRAS* in the R3 population at passages 63, 73 and 76 (23, 33 and 36 passages respectively of continuous culture in 1 μm PD173074) increased, indicating a selective advantage (supplementary material, Figure S3A). Its role in acquired resistance was confirmed by drug sensitivity assay of parental RT112 cells ectopically expressing mutant HRAS [protein] (G12V) and R3 single‐cell clones with wild type or mutant *HRAS* (G12S) (supplementary material, Figure S3B).

### EGFR, ERBB2, ERBB3 and MET have limited effect on resistance

**Figure 4:**
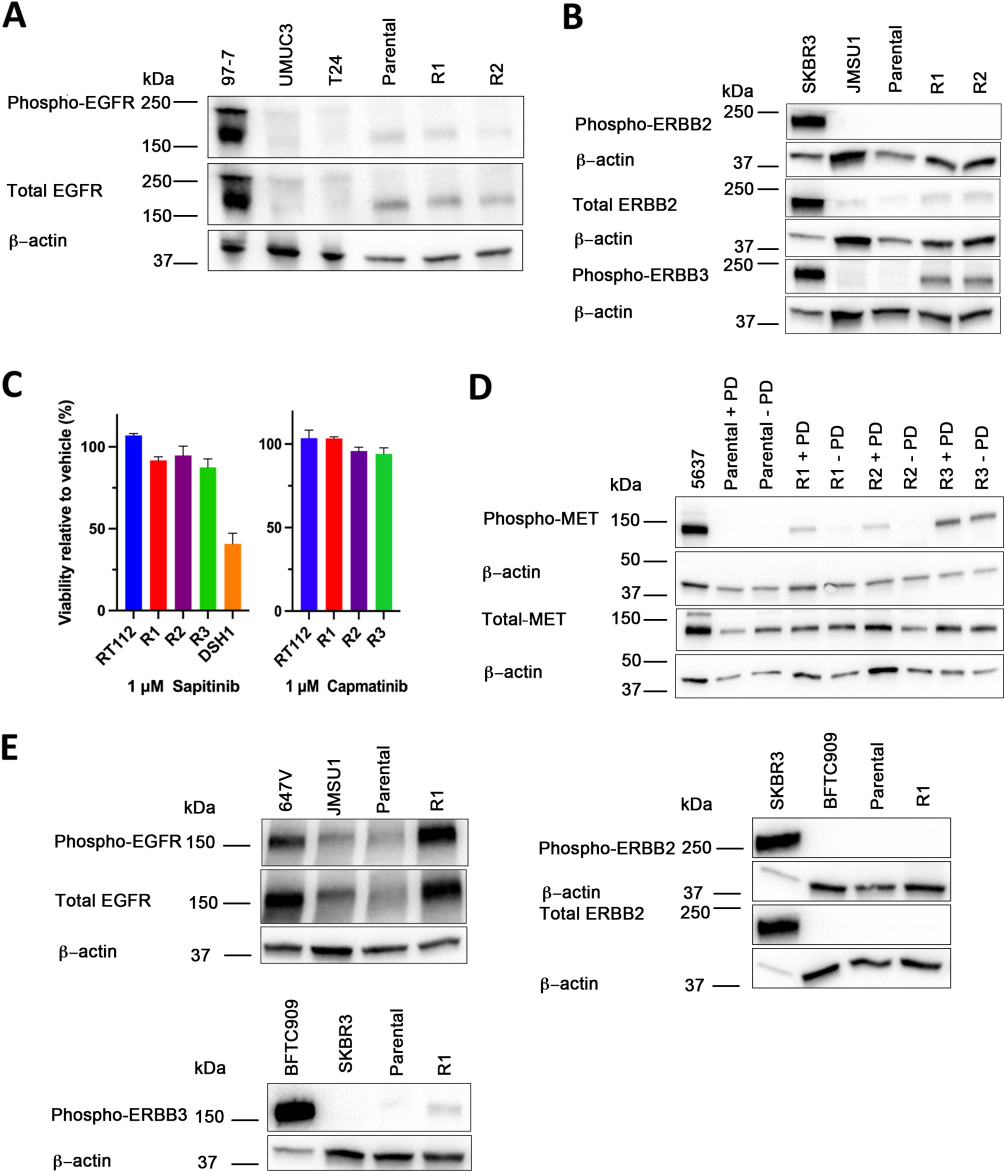
Changes in expression of receptor tyrosine kinases. (A–D) RT112 and resistant derivatives. (A) Immunoblot showing EGFR and phospho‐EGFR. (B) Immunoblots showing ERBB2, phospho‐ERBB2 and phospho‐ERBB3. (C) Viability of RT112 and PD173074 resistant derivatives in 1 μm sapitinib and 1 μm capmatinib. (D) Immunoblot showing MET and phospho‐MET. (E) Expression of EGFR, phospho‐EGFR, ERBB2, phospho‐ERBB2 and phospho‐ERBB3 in RT4 and resistant derivatives. For immunoblots, 97‐7, UM‐UC3, T24, JMSU1 and SKBR cell lysates were used as positive or negative controls. β‐actin was used as a loading control. Immunoblots were conducted at least twice and representative examples shown.

Amplification or mutation of *EGFR*, *ERBB2* and *ERBB3* genes is found in MIBC [15]. Increased signalling via EGFR has been reported as a survival pathway in RT112 following short‐term PD173074 treatment [25], and activation of ERBB2/3 has been reported in RT112 with acquired resistance to infigratinib [27]. Expression of total pEGFR was unchanged in RT112 R1 and R2 (Figure 4A). ERBB2 expression and phosphorylation was not upregulated (Figure 4B), though there was a slight increase in mRNA during acute treatment (*p* = 0.055). Increased pERBB3 was present in RT112 R1 and R2 (Figure 4B). However, treatment with the EGFR/ERBB inhibitor sapitinib did not alter viability, either as a single agent (Figure 4C) or in combination with 1 μm PD173074 (not shown).

In addition to other EGFR family members, MET can heterodimerise with ERBB3 to induce intracellular signalling [31] and is a known inducer of EMT in bladder cancer cells [32]. Examination of levels of MET and pMET showed a modest increase of pMET in RT112 R1 and R2 in drug only and a larger increase in R3 both in and out of PD173074 (Figure 4D). However, none of the resistant derivatives responded to treatment with the MET inhibitor capmatinib (Figure 4C).

In contrast, RT4 R1 cells showed a significant increase in the level of EGFR and pEGFR but not ERBB2 or ERBB3 (Figure 4E). Since RT4 R1 cultured out of drug for ≥4 passages regained sensitivity to PD173074 (Figure 3D), this suggested that the changes in EGFR/pEGFR levels were due to gene expression modulation rather than a genomic alteration. Indeed, copy number analysis showed no amplification of *EGFR*, and no mutations were found by sequencing of *EGFR* using a NGS assay that covered the majority of *EGFR* driver mutations in exons 18–21 [33]. However, despite the major change in EGFR expression, erlotinib treatment was not effective alone or in combination with PD173074.

### IGF1R as a mechanism of resistance

**Figure 5:**
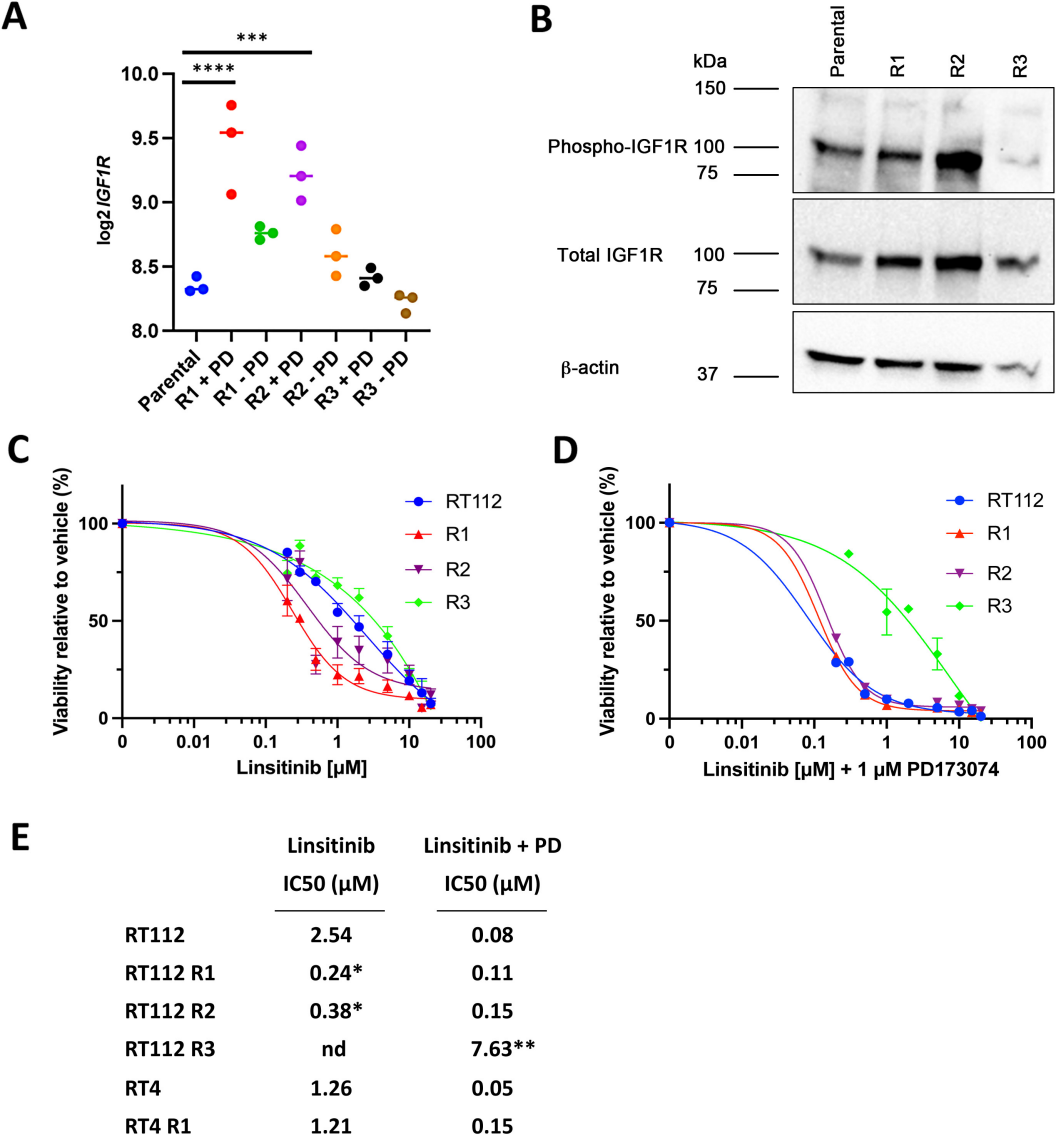
Signalling via IGF1R. (A) *IGF1R* mRNA expression in RT112 and resistant derivatives. (B) Expression of IGF1R and phospho‐IGF1R in RT112 determined by immunoblotting. (C and D) Effects of linsitinib alone and in combination with 1 μm PD173074 on RT112 and resistant derivatives. (E) IC_50_ values. In (A), One‐way ANOVA with Tukey's multiple comparisons test. ****p < 0.0001, ***p < 0.001. In (C) and (D), graphs show mean ± SEM of two to four experiments unless SEM was smaller than the symbol. (E) IC_50_ values. *p < 0.05, **p < 0.01 compared to parental RT112, nd = value not determined.

We examined the mRNA expression of a panel of receptor tyrosine kinases in addition to those previously implicated in mediating FGFR inhibitor resistance. *INSR* was slightly increased in RT112 R1 (*p* = 0.03) but not in R2. Levels of IGF1R mRNA and protein were higher in both RT112 R1 and R2 in the presence of drug and reverted in the absence of drug (Figure 5A,B). IGF1R can heterodimerise with EGFR to promote resistance to EGFR inhibitors [34, 35]. Viability assays with linsitinib (OSI‐906), a small‐molecule ATP‐competitive inhibitor of IGF1R and insulin receptor, showed that R1 and R2 were more sensitive than parental RT112 (Figure 5C,E). When combined with 1 μm PD173074, parental RT112, R1 and R2 showed similar sensitivity (Figure 5D,E). We conclude that IGF1R has a role in mediating resistance to PD173074 in R1 and R2, which can be overcome by IGF1R inhibition. R3 showed similar sensitivity to parental RT112 in linsitinib alone and was resistant to the linsitinib–PD173074 combination. *IGF1R* mRNA expression was not elevated in RT4 R1, which showed no differential sensitivity to linsitinib (Figure 5E).

### Transcriptome analysis of resistant derivatives

Because resistance mechanisms described previously in both long‐term FGFR‐resistant RT112 and short‐term escape from acute FGFR treatment [25, 27] could not fully explain the resistant phenotypes, transcriptome analysis was undertaken. Transcriptomes of RT112 and RT4 parental cells, RT112 cells acutely treated with PD173074 (24 h), RT112 and RT4 resistant derivatives cultured with drug and four to six passages without drug were analysed.

Differentially expressed genes (DEGs) were identified by pairwise comparisons (LIMMA test, FDR 0.01) (supplementary material, Tables S4–6). Unsupervised hierarchical clustering of all 7,303 DEGs identified in RT112 revealed close clustering of R1 and R2 cells cultured in drug. When cultured without drug, both variants showed a closer relationship to parental cells. R3 cells showed relatively similar profiles both with and without drug, and these were most closely related to those of parental cells and R1 and R2 cultured without drug (supplementary material, Figure S4A). Similarly, using hierarchical clustering with 2,760 DEGs, RT4 parental and R1 samples cultured without drug clustered together and separately from R1 in drug (supplementary material, Figure S5).

Dysregulated pathways were identified by GO and GSEA (supplementary material, Tables S7–S10). When compared with parental RT112, R1 and R2 showed enrichment for categories related to cell adhesion and cell motility, IFNγ response and hippo signalling and a strong EMT signature (supplementary material, Tables S7, S8 and Figure S4B,C). Processes related to active proliferation, including RNA processing, lipid metabolism and cellular respiration, were reduced, as were genes related to PPARG signalling. Similarly, RT4 R1 showed enrichment of genes involved in cell adhesion and motility, EMT and IFN responses and underrepresentation of genes involved in lipid metabolism and PPARG signalling (supplementary material, Tables S9, S10).

Du *et al* reported that knockdown of FGFR3 in RT112 resulted in downregulation of a set of 33 genes involved in fatty acid and sterol biosynthesis and metabolism [36]. Unsupervised hierarchical cluster analysis of this gene set showed significant downregulation in RT112 acutely treated with PD173074 and generally low expression in R1 and R2 in drug (supplementary material, Figure S4D), indicating that the mechanism of resistance in R1 and R2 did not re‐establish expression of lipogenic genes. In R3, however, expression of these genes was reactivated, although levels were lower than in untreated parental RT112, consistent with the conclusion of Du *et al* that FGFR3 induces lipogenesis mainly through PI3K/mTOR signalling rather than via RAS/ERK/MEK [36].

Modulation of chromatin state associated with upregulation or dependency on histone demethylases KDM5A and KDM6A or histone methyl transferases SETDB1/2 has been implicated in the generation of a reversible drug‐tolerant state [37, 38, 39]. As found here, IGF1R is reported to be upregulated in so‐called drug‐tolerant persister cells (DTPs) and a relationship between upregulated KDM5A and IGF1R is reported [37]. We assessed the expression of a range of chromatin modifiers and found upregulated expression of *KDM6A* in RT112 R1 and R2 and in RT4 R1 (supplementary data, Figure S6). However, no differential sensitivity of parental and resistant cells was found to the JMJD3/KDM6A inhibitor GSK‐J4 [40] either alone or in combination with PD173074 (not shown).

We assessed the expression of other markers associated with DTPs. In addition to an EMT‐like phenotype and upregulation of the phospholipid glutathione peroxidase *GPX4* [41, 42], the stem cell markers *CD24* and *IGFBP3* [37] and peroxiredoxin 6 (*PRDX6*) involved in oxidative stress management [43] were upregulated in RT112 R1 and R2 (supplemental material, Figure S7). RT4 R1 showed no or only marginal changes in these genes.

Bladder cancers can be classified into two major molecular subtypes, ‘luminal’ and ‘basal‐squamous’ [44]. Both RT112 and RT4 have been classified as luminal [45]. We assessed a basal‐squamous differentiation signature in parental cells and resistant derivatives and found no differences. An FGFR3‐related signature was reduced in resistant cells from both cell lines. This showed partial reversal in RT112 when cells were cultured without drug (supplementary material, Figure S8A). In RT4, resistant cells showed further decrease in this signature when cultured without drug (supplementary material, Figure S8B). A PPARG signalling‐related signature [46] was strongly downregulated in both RT112 resistant derivatives and showed upregulation following removal of drug (supplementary material, Figure S8C). A slight but non‐significant reduction in the expression of this signature was apparent in RT4 R1. Because PPARG signalling is implicated in the regulation of urothelial differentiation [47], we examined a urothelial differentiation signature in RT112 and found this to be reduced in R1 and R2 (*p* = 0.0141 and 0.0034 respectively).

### YAP/TAZ activation in resistant cells

**Figure 6:**
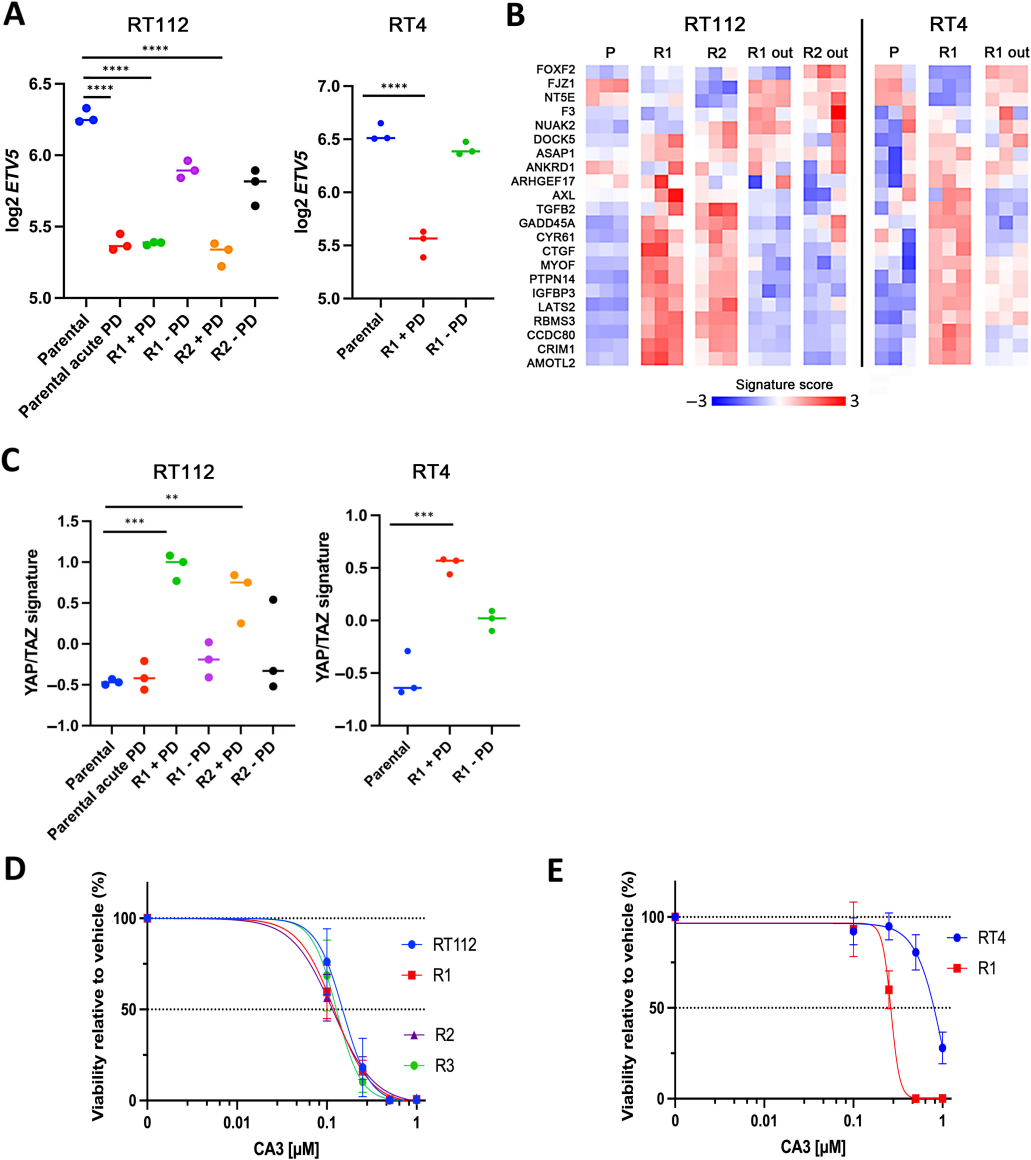
Expression changes associated with FGFR3 inhibition and upregulation of YAP/TAZ signalling in RT112, RT4 and resistant derivatives. (A) mRNA expression of *ETV5*. (B) Heatmap of *z*‐scores for YAP/TAZ signature genes [54]. (C) YAP/TAZ signature scores. (D and E) Dose response curves for CA3. Curves show means and SEM of three to seven assays. In (A) and (C), one‐way ANOVA with Tukey's multiple comparisons test. ****p < 0.0001, ***p < 0.001, **p < 0.01.

The Hippo pathway and its transcriptional coactivator Yes‐associated protein 1 (YAP1) is implicated in bladder cancer tumorigenesis [48, 49, 50] and resistance to therapy [51, 52, 53]. Because Hippo signalling genes had been identified as upregulated in RT112 resistant cells, we examined this further. Previously we showed that ETV5 is upregulated downstream of point‐mutated *FGFR3* in urothelial tumour cells, leading to upregulation of TAZ (WWTR1) and implicating YAP/TAZ signalling in the mediation of some of the oncogenic effects of FGFR3 [48]. As expected, *ETV5* was downregulated following acute FGFR3 inhibition in RT112 and remained at low levels in RT112 and RT4 resistant lines in the presence of PD173074 (Figure 6A). However, all resistant derivatives showed upregulation of a YAP/TAZ target signature [54] (Figure 6B,C), suggesting regulation via a distinct mechanism not downstream of ETV5.

We examined the effects of the YAP1 inhibitor CA3 [55] in both cell lines and resistant derivatives. No differences in sensitivity were detected between RT112 and its PD173074‐resistant derivatives (Figure 6D). When challenged with CA3 in the presence or absence of PD173074, parental RT4 cells were insensitive. However, RT4 R1 were sensitive to CA3 (*p* < 0.01), and this reverted with continued culture in the absence of PD173074 (Figure 6E).

## Discussion

Although results from trials of FGFR inhibitors in patients with FGFR3 alterations are encouraging, responses are short‐lived. In the BLC2001 study of erdafitinib in patients with locally advanced and unresectable or metastatic urothelial carcinoma, although the objective response rate was 40%, median duration of response was only 6 months [56], indicating that resistance is acquired rapidly. Because all patients in the study had one or more FGFR alterations, it is also clear that despite the presence of the target, many tumours are intrinsically resistant. Thus, improved understanding of both intrinsic and acquired resistance to FGFR3 inhibition is urgently required.

We explored potential mechanisms of acquired resistance in two urothelial carcinoma cell lines that contain FGFR3–TACC3 fusion proteins, both of which showed good initial response to treatment [17]. A range of mechanisms is reported to allow escape from FGFR inhibition either following short‐term exposure or in cells with long‐term acquired resistance [23, 24, 25, 26, 27, 29, 57]. By examining several lines of resistant derivatives, we demonstrated that both genetic and epigenetic mechanisms of resistance can arise.

In RT112 R3, resistance was due to acquisition of an *HRAS* mutation. This corroborates our previous study, which showed that bladder tumour cell lines containing mutant *RAS* were intrinsically resistant to FGFR inhibition [17] and a recent finding of resistance due to overexpressed wild type *HRAS* in FGFR‐dependent cell lines, including RT112 [28]. Synergy between RAS and PI3K pathways in overcoming FGFR inhibition was also reported by Wang *et al* [57]. RT112 cells carry only one copy of *HRAS* due to a deletion of the region of chromosome 11 that includes *HRAS* (this study and [58, 59]). Although DNA samples were not collected during derivation of the resistant lines, it is likely that the *HRAS* mutation arose relatively early as this derivative stabilised earlier than the others. Nevertheless, despite continued selection, around 10% of R3 cells remained *HRAS* wild type even after 36 passages of continuous culture in drug, and these presumably survived using another mechanism. It is notable that no *FGFR3* gatekeeper mutations were found.

The reversal of mesenchymal morphology and resistance after removal of drug in all other resistant derivatives indicates changes in gene expression or signalling via alternative pathways. Although we found changes in expression and/or phosphorylation of several receptor tyrosine kinases that have been implicated in providing escape from FGFR inhibition (EGFR, ERBB2, ERBB3 and MET), none were associated with sensitivity to relevant inhibitors.

All resistant derivatives showed upregulated expression of YAP/TAZ targets, and in RT4 resistant cells this was associated with sensitivity to the YAP inhibitor CA3, which suggests a potential approach to therapy. However, this was not the case in RT112 resistant cells. This and the lack of response to EGFR inhibition in RT4 R1, despite the presence of major upregulation and activation of EGFR, suggest the caveat that, similar to the finding that FGFR‐mutant tumours do not all respond to FGFR inhibition, the presence of a putative predictive biomarker in many cases does not predict response.

Upregulated expression of IGF1R in RT112 R1 and R2 was associated with sensitivity to linsitinib (OSI‐906), a small‐molecule ATP‐competitive inhibitor of IGF1R and insulin receptor. Inhibition of IGF1R in combination with gefitinib was reported to eliminate the emergence of DTPs in the EGFR‐dependent lung cell line PC9 and to restore the reduced H3K4 methylation that results from activity of KDM5A in this system [37]. The reversible state, IGF1R sensitivity and upregulation of KDM6A in RT112 resistant cells, suggests that these resistant derivatives represent proliferating DTPs. However, despite upregulation of KDM6A in RT4 R1, these cells did not show upregulation of other markers of DTPs. It is clear that considerable heterogeneity in mechanisms exists within the DTP state both within and between cell lines and in a large‐scale drug screen of erlotinib‐resistant persister‐derived cells in the PC9 system, no single category of drug was identified to which all were sensitive [60]. It is also apparent that the state in early non‐dividing persister cells differs from that in emergent cycling cells [61]. This heterogeneity and the commonality of the persister state pose a considerable challenge for personalised therapy and motivation for further work.

In conclusion, our findings indicate diversity in resistance mechanisms in the response of urothelial cells to FGFR3 inhibition. Collection of tissue samples from patients in relapse is now urgently needed to determine whether any of these mechanisms are observed in the clinic. Although our data identify drug combinations that are effective in some resistant cells, they also identify significant problems in identifying effective predictive biomarkers for use in the relapsed disease setting. However, the reversibility of the resistant state in most FGFR‐resistant cells may suggest resumption of treatment after disease relapse.

## Author contributions statement

MK obtained funding. GP, HM, MD, ZK, JB, CH, FP and EB carried out experimental work.

CH, OA, GP and MK carried out data analysis. JB, GP and MK wrote the manuscript, and all authors revised the manuscript and approved the submitted version.

## Supporting information


**Supplementary materials** **and methods**

**Figure S1.** Expression of markers of epithelial–mesenchymal transition
**Figure S2.** Copy number analysis of RT112 and derivatives R1 and R3
**Figure S3.** Detection of *HRAS* (G12S) mutation in RT112 R3
**Figure S4.** Expression features of RT112 and PD173074‐resistant derivatives
**Figure S5.** Expression features of RT4 and PD173074‐resistant derivative R1
**Figure S6.** Expression of *KDM6A* mRNA in RT112, RT4 and PD173074‐resistant derivatives
**Figure S7.** Expression of reported markers of a drug‐tolerant state (*CD24*, *IGFBP3* and *PRDX6*) in RT112 and PD173074‐resistant derivatives
**Figure S8.** Expression of *FGFR3*, an FGFR3 signature and a PPAR gamma‐related signature in RT112, RT4 and PD173074‐resistant derivatives
**Table S1.** STR profiles of RT112 and RT4
**Table S2.** Mutations identified in RT112 R3
**Table S3.** Copy number alterations in RT112 parental and derivative cell lines
**Table S4.** Differentially expressed genes (limma test, FDR 0.01) in comparisons of RT112 and RT4 experimental conditions
**Table S5.** Information on genes significantly differentially expressed between RT112 parental and resistant lines
**Table S6.** Information on genes significantly differentially expressed between RT4 parental and resistant lines
**Table S7.** GO analysis of RT112 parental and resistant lines
**Table S8.** GSEA analysis of parental RT112 and resistant lines
**Table S9.** GO analysis of parental RT4 and resistant line
**Table S10.** GSEA analysis of parental RT4 and resistant line

## Data Availability

Microarray data are available at the Gene Expression Omnibus under Accession No. GSE201395 (https://www.ncbi.nlm.nih.gov/geo/query/acc.cgi?acc=GSE201395).
