# Supplementary material for: Development of resistance to FGFR inhibition in urothelial carcinoma via multiple pathways in vitro
Source: J Pathol. 2022 Dec 13;259(2):220–32. doi: 10.1002/path.6034 (PMC10107504; doi:10.1002/path.6034)
Supplement: Supplementary file 1 — Supplementary materials and methods [file PATH-259-220-s002.docx]

**Development of resistance to FGFR inhibition in urothelial carcinoma via multiple pathways *in vitro***

GA Pettitt *et al. J Pathol* <https://doi.org/10.1002/path.6034>

**Supplementary materials and methods**

Reference numbers refer to the main text list

**Cell culture**

Parental cell lines were STR-profiled in house using PowerPlex16 reagents (Promega, Madison, WI, USA) and used within 10 passages of this stock; derivatives were not STR-tested directly but compared to parental lines by next-generation sequencing (NGS). STR profiles are provided in supplementary material, Table S1. Cells were regularly tested for mycoplasma using an in-house service. Single-cell clones of RT112 were generated by plating 96-well dishes at a density of 0.5, or 1 cell per well. Wells in which only one colony developed were expanded for analysis.

Stable ectopic expression of HRAS [protein] G12V was achieved by transducing RT112 with retrovirus produced in Phoenix-AMPHO cells from pLXSN-HRAS V12 (a gift from Julian Downward, plasmid no. 39516; Addgene, Watertown, MA, USA [65]) followed by 2 weeks of selection with G418.

**Cell viability assays**

CellTiter-Blue (Promega) reagent was used according to the manufacturer’s instructions to measure cell viability. Cells were seeded into 96-well plates, 5 or 10 wells per treatment, at a density that would give 80% confluence at the end of the assay in the untreated control. Cells were treated with drug or vehicle (0.1% DMSO) 24 h after seeding and incubated for 72 h, at which point medium was changed and cells were incubated with treatment for a further 48 h. Fluorescence was measured using a Berthold Mithras LB 940 Multimode microplate reader, (Berthold Technologies, Bad Wildbad, Germany). Results were normalised to the vehicle control and dose response curves, and IC50s were calculated in GraphPad Prism 9 (GraphPad, San Diego, CA, USA). Assays were performed at least twice.

**Immunoblotting**

Cells at 70–80% confluence were lysed in RIPA buffer (PBS containing 1% Triton X100, 1 mM EDTA, 0.5% sodium deoxycholate, 0.1% SDS, protease and phosphatase inhibitor cocktails [P8340, P5726; Sigma-Aldrich, St. Louis, MO, USA]), and 30 μg of cleared lysate was run on 7.5% or Any kD Bio-Rad MiniPROTEAN TGX precast polyacrylamide gels (Bio-Rad, Hercules, CA, USA) according to the manufacturer’s instructions. Proteins were transferred to nitrocellulose for immunoblot analysis. Bound primary antibody was detected using horseradish peroxidase-linked secondary antibodies, either anti-rabbit IgG (1:3000; SouthernBiotech, Birmingham, AL, USA) or anti-mouse IgG (Bio-Rad), and Luminata Forte detection reagent (MilliporeSigma, Burlington, MA, USA). Chemiluminescence images were captured and analysed using Image Lab software (Bio-Rad).

**mRNA expression arrays**

RNA was extracted using an RNeasy Mini Kit (Qiagen, Hilden, Germany) according to the manufacturer’s protocol. RNA (10 μg) was treated with 3 U of DNase I in DNase reaction buffer containing 40 U RNase OUT™ (all reagents from Life Technologies, Carlsbad, CA, USA) in a total volume of 34 μl for 15 min at room temperature. The reaction was inhibited by the addition of 4 μl of 25 mM EDTA, and RNA was purified with an RNeasy Mini Kit (Qiagen) using the RNA Clean-up protocol according to the manufacturer’s protocol. Microarray analysis was conducted by Hologic/Tepnel (Livingston, West Lothian, UK) as follows: total RNA was amplified using the Affymetrix GeneChip® WT PLUS Reagent Kit (Affymetrix, Santa Clara, CA, USA) according to the manufacturer’s instructions. The resulting cDNA was quantified using optical density (OD_260_) (NanoDrop, Thermo Fisher Scientific, Waltham, MA, USA). The cDNA was normalised and hybridised onto Affymetrix Human Transcriptome 2.0 microarrays for 16 h at 45 °C. Microarrays were washed and stained using the Affymetrix GeneChip® Hybridization, Wash, and Stain Kit following the manufacturer’s instructions, using the Affymetrix GeneChip® Fluidics Station 450. Microarrays were scanned using an Affymetrix GeneChip® 7G microarray scanner. Affymetrix® Expression Console™ Software was used to examine data quality. Affymetrix HTA 2.0 CEL files were normalised as rma_sketch using apt-probeset-summarize from the Affymetrix Power Tools and HTA-2_0.r1.gene.cdf. After normalisation, the dataset was loaded into the R2: genomics analysis and visualisation platform (http://r2.amc.nl). R2 was used for routine data visualisation, data mining and analysis.

**Gene expression signatures**

We used gene lists associated with FGFR3-related gene expression [64], urothelial differentiation (*KLF5, PPARG, RXRA, ELF3, FOXA1, GATA3, TP63, GRHL2, GRHL3*), PPARG signalling [47], EMT [63],YAP/TAZ targets [55] and basal-squamous (*CD44, DSC2, DSC3, DSG1, DSG3, EGFR, ITGA6, KRT14, KRT5, KRT6, PI3, SPRR1A, SPRR1B, SPRR2A, SPRR2B*) urothelial tumours. Signature zscores for these gene sets were derived for each sample, weighted where up- and downregulated genes were included.

**Gene Ontology and Gene Set Enrichment Analysis**

Significantly upregulated genes (LIMMA test, false discovery rate *p* value < 0.01) in each pairwise comparison was used as input for Gene Ontology (GO) biological process analysis in R2. A cut-off of nominal *p* value < 0.05 was implemented. Gene Set Enrichment Analysis (GSEA) version 3.0 was carried out using all genes; parental and resistant derivatives were assigned as phenotypes and permuted 1,000 times, and the test dataset was collapsed to gene symbols and run against gene sets in the Hallmarks database (version 7.1).

**Whole exome sequencing**

DNA used for exome sequencing and copy number analysis was purified from cells using a Gentra Puregene Kit (Qiagen) following the manufacturer’s cultured cells protocol. DNA was sonicated with a Covaris S2 sonicator (Covaris, Woburn, MA, USA). Libraries were generated and enriched for exomic regions using the SureSelect^XT^ Human All Exon V6 capture library (Agilent, Santa Clara, CA, USA) following the manufacturer’s protocols. NGS was performed by the Leeds Institute of Medical Research Next Generation Sequencing Facility. Base calling and quality control were performed using Illumina’s Real Time Analysis software (Illumina, San Diego, CA, USA) with standard settings. Sequence files were QC checked using FastQC (version 0.10.0) before preprocessing. Adapter contamination and low-quality read ends (< 20) were trimmed using cutadapt 1.3 and fastq-tools 0.8. Any read in which either of a pair had a length less than 19 was removed from subsequent analysis. Read mapping and genotype calling alignment was performed using BWA 0.7.10 mem GRch38 reference. Duplicate reads were removed using the Picard version 1.56 MarkDuplicates program. Local realignment around indels was performed using the GATK version 1.3 RealignerTargetCreator and IndelRealigner in Smith–Waterman mode with reference to dbSNP version 132. The Picard version 1.56 FixMateInformation program was used to ensure that all mate-pair information was in sync between each read and its mate following local realignment. Base quality scores were recalibrated using GATK version 1.3 CountCovariates and TableRecalibration with reference to dbSNP version 132. BAM files were sorted and then indexed using SAMtools index. Somatic Variant Analysis Pileup files (created using SAMtools mpileup, with parameters – d 5000 and q- 20) were used as input to VarScan2 version 2.3.5. This was used in somatic mode, with a strand filter, to identify somatic single nucleotide variations (SNVs) and small insertions and deletions (indels). Results were then processed using processSomatic to extract the somatic mutations with the specification that zero reads were to support the variant in the normal sample. SNVs and indels were identified in RT112 R1 and RT112 R3, with parental RT112 as the normal sample. To be called, a mutation needed to satisfy the following two criteria: (1) a variant allele frequency of greater than 20% and (2) an absence of the mutation in RT112 parental. High-confidence somatic variant and indel calls were converted to mutation annotation format (MAF) using Variant Effect Predictor (VEP) version 81 via Ensembl Virtual Machine.

**Copy number analysis**

Copy number alterations were examined using whole exome sequencing data. Following the generation of BAM files (see above), and ngCGH was used to compare the number of read counts between the cell line and blood or parental samples using a window size of 1,000. The Nexus Copy Number software package (BioDiscovery, El Segundo, CA, USA) was used to conduct GC correction and copy number calling using the FASST2 segmentation algorithm. Segmentation was conducted with a significance threshold of 1.0E-5, with a requirement of at least three probes per segment and a maximum probe spacing of 1,000 between adjacent probes before breaking a segment. The log2 ratio thresholds were set as follows: single copy gain 0.25, single copy loss −0.25, two or more copy gains 1.25, homozygous loss −1.25.

**PCR**

DNA used for Sanger sequencing of *HRAS* exon 1 was purified using a QIAamp DNA Mini kit (Qiagen) following the blood or body fluids spin protocol. Standard PCR was performed, with each reaction containing 1× AmpliTaq Gold® 360 buffer (Thermo Fisher Scientific), 2.5 mM MgCl_2_, 0.2 μM each forward and reverse primers (forward 5’-CAGGAGACCCTGTAGGAGGA-3’, reverse 5’-TCGTCCACAAAATGGTTCTG-3’), 1 mM dNTPs, 1 U AmpliTaq Gold® 360 DNA Polymerase and 20 ng DNA in a total reaction volume of 25 μl. PCR cycling conditions were as follows: 95 °C for 5 min, 35 cycles of 95 °C for 30 s, 60 °C for 30 s and 72 °C for 30 s, followed by a final extension step of 72 °C for 10 min.

**Sanger sequencing**

PCR products were treated with 1 μl ExoProStar (Illumina) and sequenced using the BigDye™ Terminator version 1.1 Cycle Sequencing Kit (Applied Biosystems, Waltham, MA, USA) according to the manufacturer’s instructions using the same *HRAS* primers as used for standard PCR. The products were analysed on an ABI 3130xl Genetic Analyzer.

**SNaPshot analysis**

Cell line DNA for SNaPshot analysis was purified and amplified by PCR following the same methodology as for sequencing. For SNaPshot analysis of single cell clones, colonies in 96-well plates were lysed with 25 μl DirectPCR reagent (Viagen Biotech, Los Angeles, CA, USA) containing Proteinase K according to the manufacturer’s protocol, and 2 μl was used for touchdown PCR with GoTaq Hot Start polymerase (Promega) in a 20 μl reaction using the *HRAS* primers shown above. Cycling conditions were as follows: 94 °C for 3 min, 3 cycles of 94 °C for 15 s, 64 °C for 30 s, 70 °C for 150 s, 3 cycles of 94 °C for 15 s, 61 °C for 30 s, 70 °C for 150 s, 3 cycles of 94 °C for 15 s, 58 °C for 30 s, 70 °C for 150 s, 35 cycles of 94 °C for 15 s, 57 °C for 30 s, 70 °C for 150 s, followed by a final extension step of 72 °C for 5 min.

SNaPshot analysis was conducted with SAP/Exonuclease-treated PCR products and ABI PRISM SNaPshot Multiplex Kit (Applied Biosystems) according to the manufacturer’s instructions using a *HRAS* cDNA 34 base pair probe with the following sequence: 5’-T(17)CTGGTGGTGGTGGGCGCC-3’. SNaPshot products were run on an ABI 3130xl Genetic Analyzer with a 120 LIZ™ dye size standard (Applied Biosystems) and analysed using GeneMapper software.
