# Supplementary material for: Development of resistance to FGFR inhibition in urothelial carcinoma via multiple pathways in vitro
Source: J Pathol. 2022 Dec 13;259(2):220–32. doi: 10.1002/path.6034 (PMC10107504; doi:10.1002/path.6034)
Supplement: Supplementary file 2 — Figure S1. Expression of markers of epithelial–mesenchymal transition Figure S2. Copy number analysis of RT112 and derivatives R1 and R3 Figure S3. Detection of HRAS (G12S) mutation in RT112 R3 Figure S4. Expression features of RT112 and PD173074‐resistant derivatives Figure S5. Expression features of RT4 and PD173074‐resistant derivative R1 Figure S6. Expression of KDM6A mRNA in RT112, RT4 and PD173074‐resistant derivatives Figure S7. Expression of reported markers of a drug‐tolerant state (CD24, IGFBP3 and PRDX6) in RT112 and PD173074‐resistant derivatives Figure S8. Expression of FGFR3, an FGFR3 signature and a PPAR gamma‐related signature in RT112, RT4 and PD173074‐resistant derivatives Table S1. STR profiles of RT112 and RT4 Table S2. Mutations identified in RT112 R3 Table S3. Copy number alterations in RT112 parental and derivative cell lines Table S4. Differentially expressed genes (limma test, FDR 0.01) in comparisons of RT112 and RT4 experimental conditions Table S5. Information on genes significantly differentially expressed between RT112 parental and resistant lines Table S6. Information on genes significantly differentially expressed between RT4 parental and resistant lines Table S7. GO analysis of RT112 parental and resistant lines Table S8. GSEA analysis of parental RT112 and resistant lines Table S9. GO analysis of parental RT4 and resistant line Table S10. GSEA analysis of parental RT4 and resistant line [file PATH-259-220-s001.zip › path6034-sup-0002-Figures S1-S8,Tables S1,S2,S4.docx]

**Development of resistance to FGFR inhibition in urothelial carcinoma via multiple pathways *in vitro***

GA Pettitt *et al. J Pathol* <https://doi.org/10.1002/path.6034>

**Supplementary Figures S1–S8**

**Supplementary Tables S1, S2, S4**

**Supplementary Tables S3, S5–S10 (separate Excel files)**

Reference numbers refer to the main text list


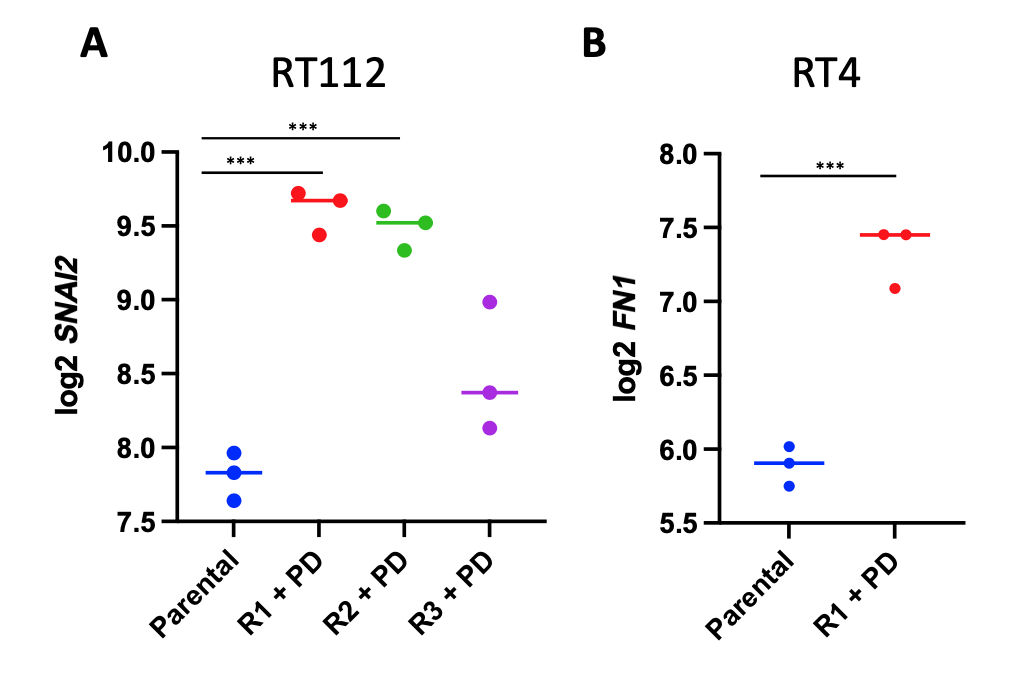


**Figure S1.** Expression of markers of epithelial-mesenchymal transition. mRNA levels of (A) Slug (*SNAI2*) in RT112 and derivatives R1, R2 and R3 and (B) fibronectin (*FN1*) in RT4 and derivative R1 in the presence of PD173074 (+ PD). *** *p*<0.001.


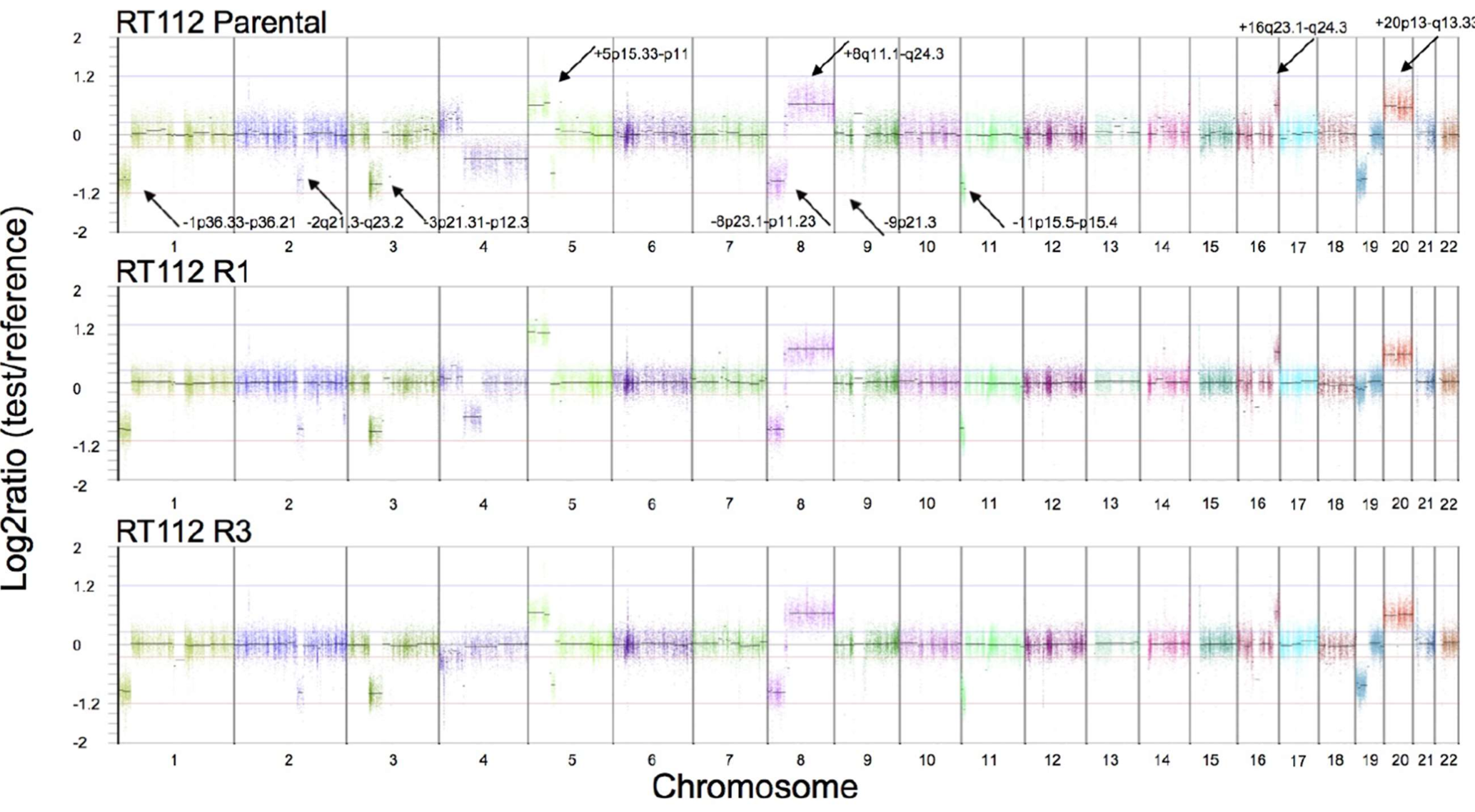


**Figure S2.** Copy number analysis of RT112 and derivatives R1 and R3. The *x*-axis corresponds to chromosomes 1 to 22. The *y*-axis corresponds to log_2_ratio (test/reference).


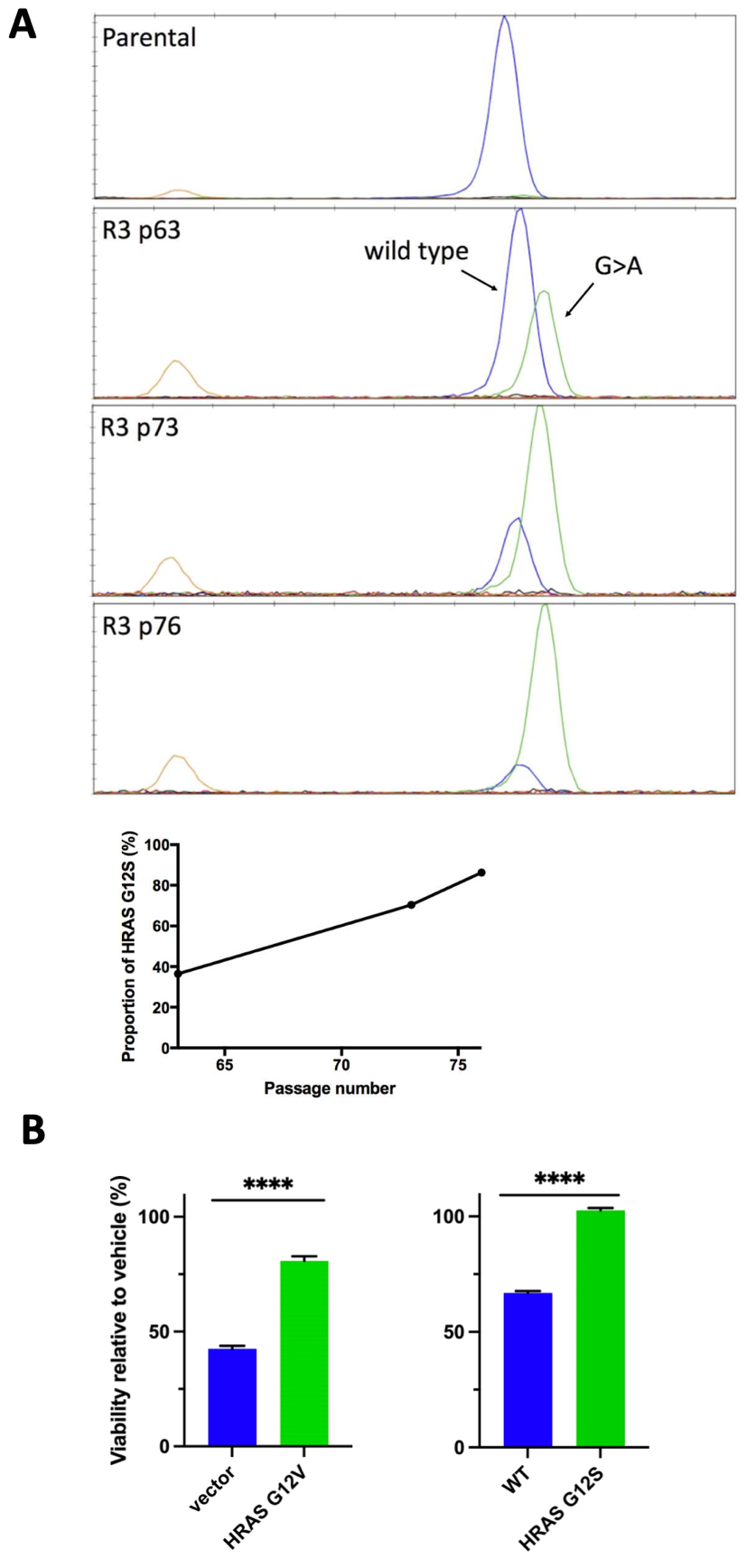


**Figure S3.** Detection of *HRAS*(G12S) mutation in RT112 R3. (A) SNaPShot analysis of *HRAS* cDNA position 34 in parental RT112 and RT112 R3 at passages 63, 73 and 76. Orange peaks represent Genescan G120-LIZ size standards, green peaks represent adenine, blue peaks represent guanine. For each RT112 R3 passage, the proportion of *HRAS*(G12S) and wild-type *HRAS* was determined by calculating the area under the green and blue curves. (B) Cell viability of RT112 retrovirally transduced to express *HRAS*(G12V) (left) and R3 single-cell clones (right). Cell viability was assayed following 120 h treatment with 1 μM PD173074 and normalised to the vehicle control. Bars show means and SEM of two (G12V) or three (G12S) experiments. **** *p*<0.0001.


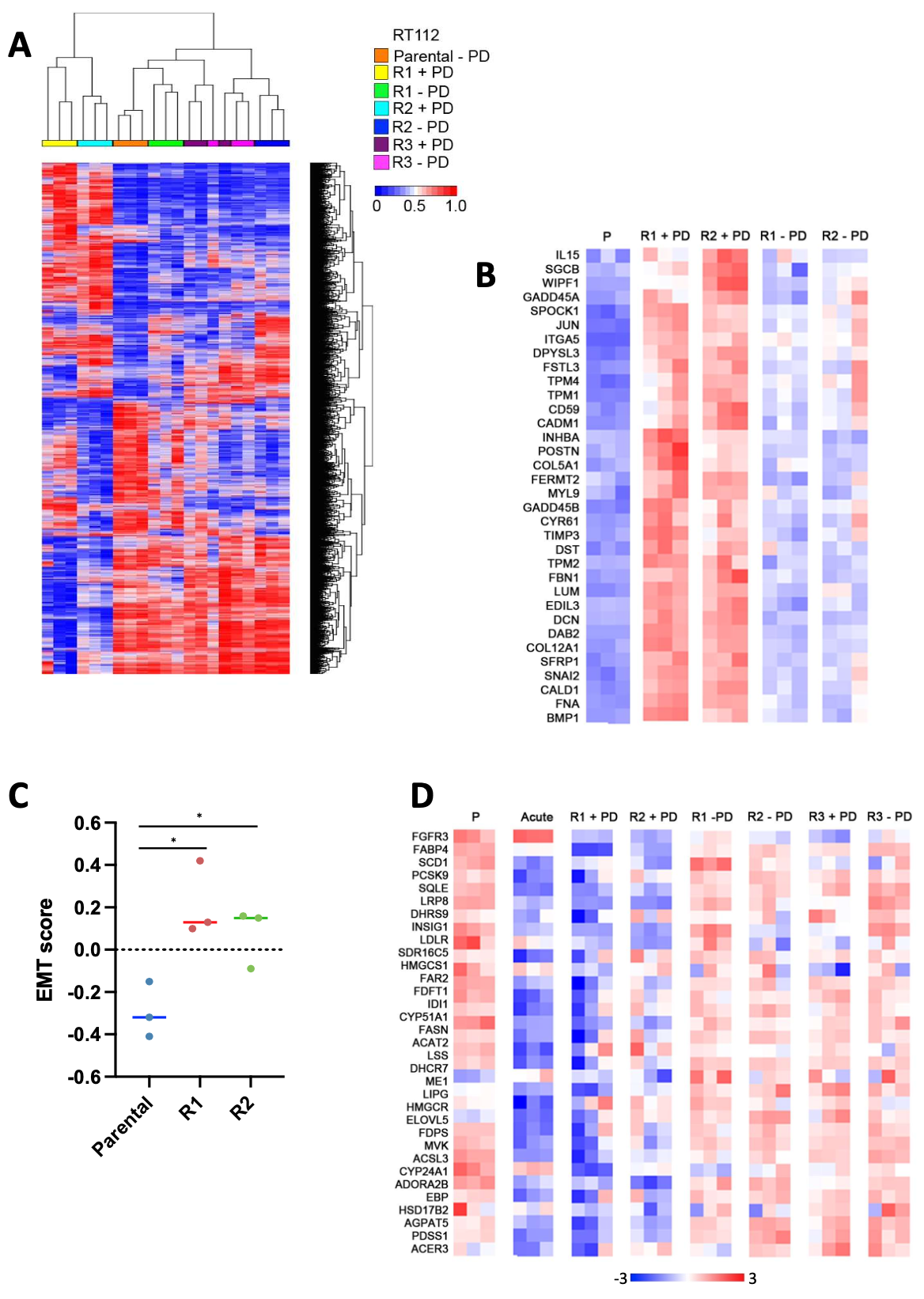


**Figure S4.** Expression features of RT112 and PD173074-resistant derivatives. (A) Hierarchical clustering of 7,303 differentially expressed genes (LIMMA test with FDR 0.01) between parental RT112 cultured in the absence of drug and other RT112 experimental conditions. Euclidean distance and complete linkage were used. Heatmap generated using tools in Partek® Genomics Suite 6.6. (B) Heatmap of *z*-scores for genes from an epithelial–mesenchymal transition signature (Hallmark EMT; MSigDB) in RT112 parental (P) and derivatives R1 and R2 in the presence of drug (+ PD) or cultured for four passages out of drug (-PD). (C) EMT signature in parental RT112, R1 and R2 resistant derivatives [1]. * *p* < 0.05. (D) Heatmap of *z*-scores for genes involved in fatty acid and sterol biosynthesis and metabolism related to expression of FGFR3 [2].


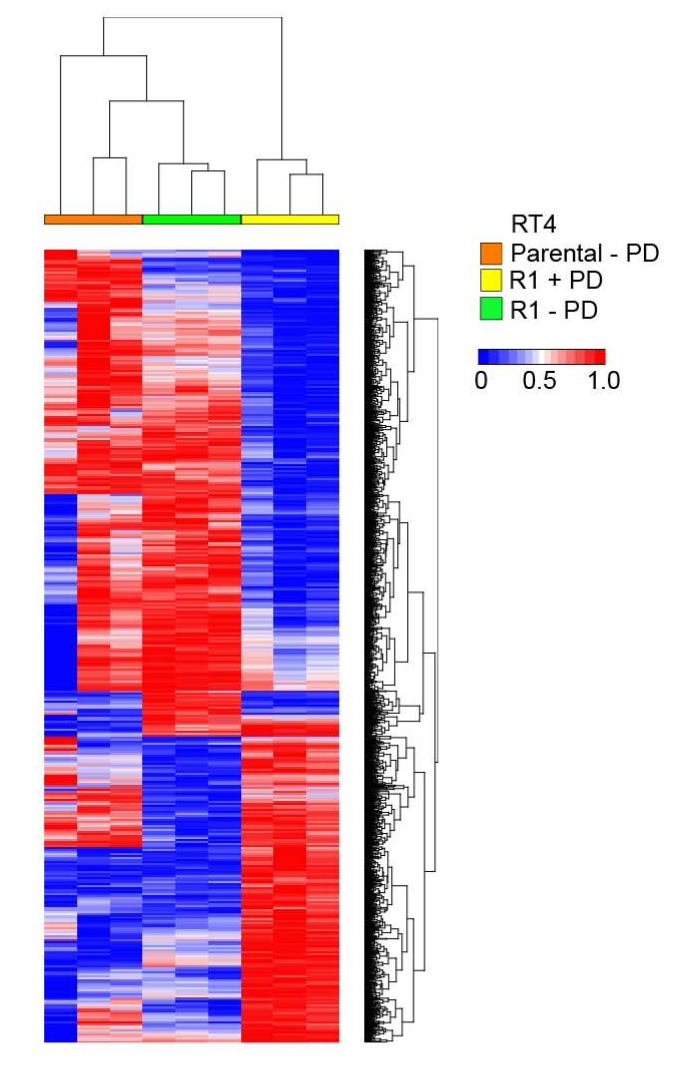


**Figure S5.** Expression features of RT4 and PD173074-resistant derivative R1. Hierarchical clustering of 2,760 differentially expressed genes (LIMMA test with FDR 0.01) between parental RT4 cultured in the absence of drug and other RT4 experimental conditions. Euclidean distance and complete linkage were used. Heatmap generated using tools in Partek® Genomics Suite 6.6.


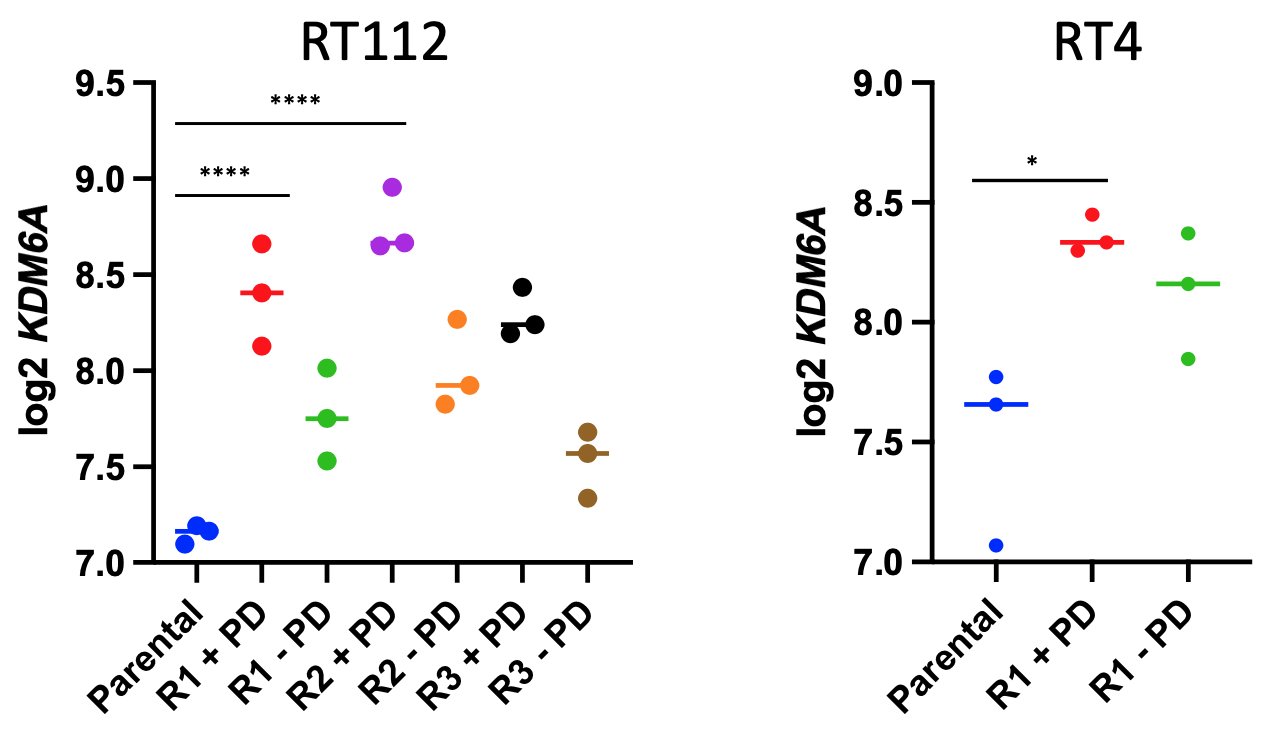


**Figure S6.** Expression of *KDM6A* mRNA in RT112, RT4 and PD173074-resistant derivatives. *****p*<0.0001, **p*<0.05.


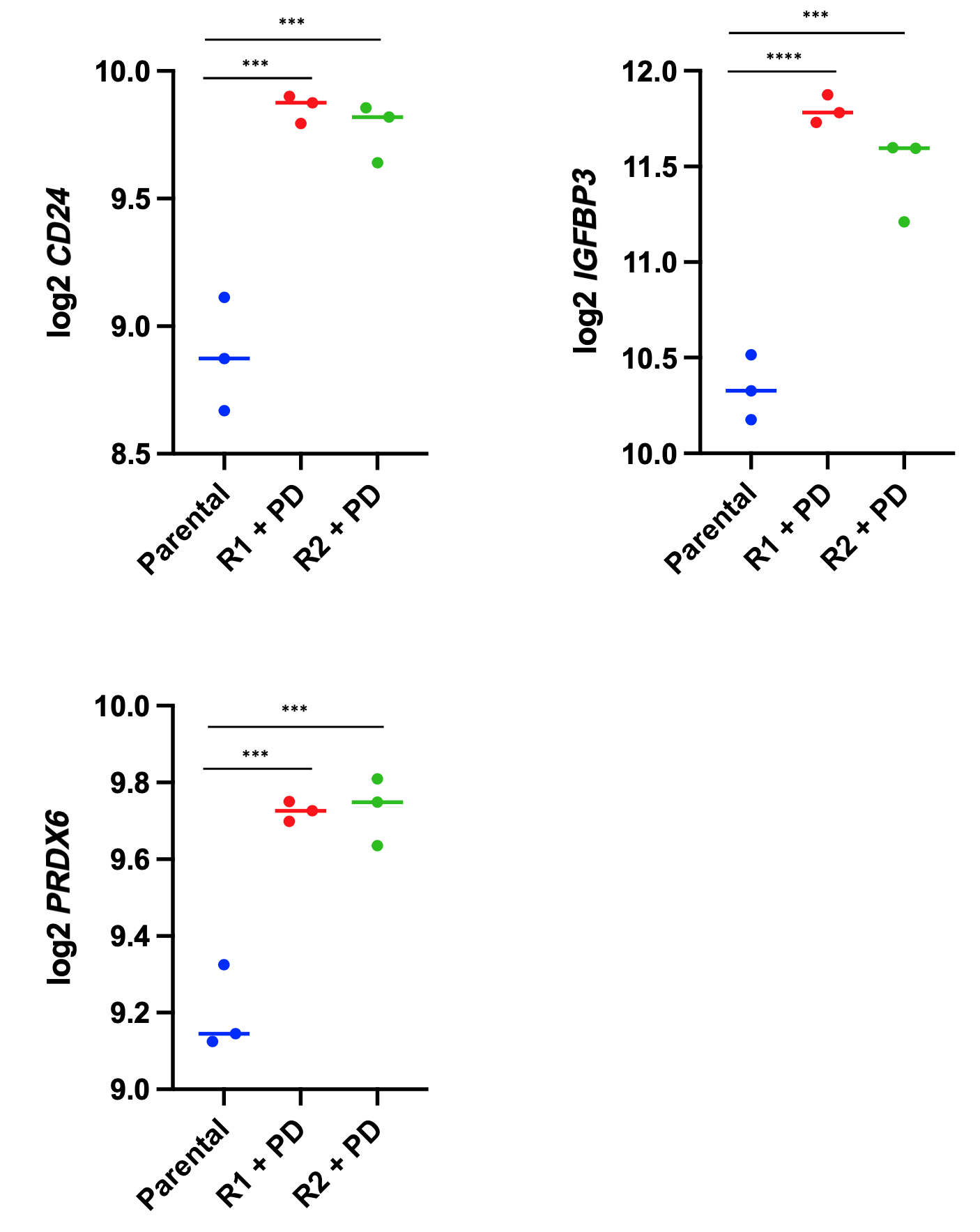


**Figure S7.** Expression of reported markers of a drug-tolerant state (*CD24*, *IGFBP3* and *PRDX6*) in RT112 and PD173074-resistant derivatives. *****p*<0.0001,****p*<0.001.


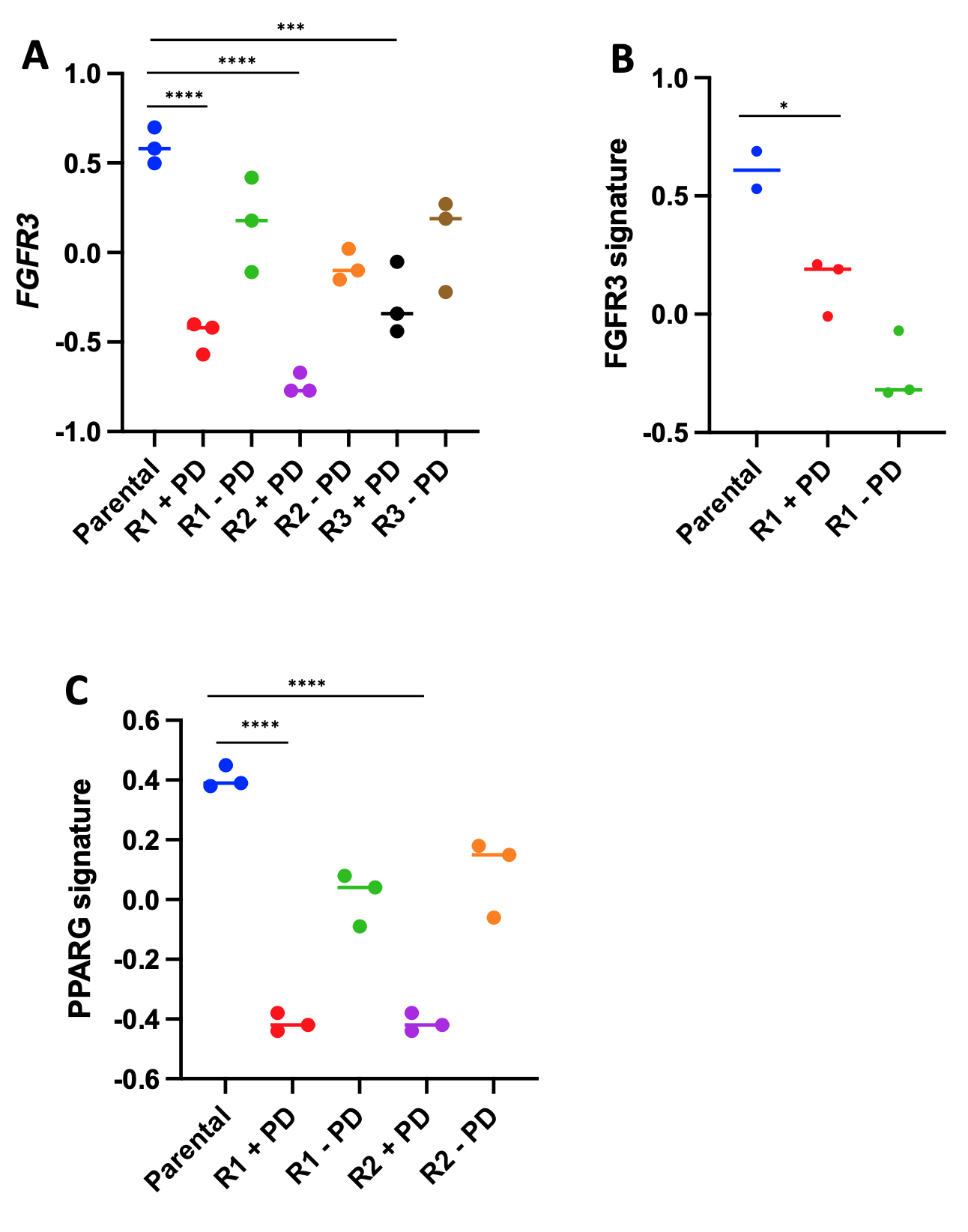


**Figure S8.** Expression of *FGFR3*, FGFR3 signature and PPAR gamma-related signature in RT112, RT4 and PD173074-resistant derivatives. (A) *FGFR3* mRNA levels in RT112 and derivatives. (B) Expression of FGFR3-related signature [3] in RT4 and its resistant derivative. (C) Expression of PPARG-related signature [4] in RT112 and derivatives.

**Supplementary Tables S1, S2, S4**


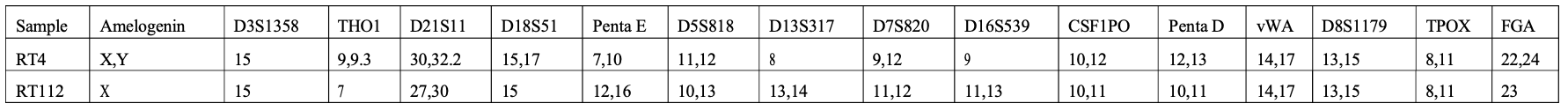


**Table S1.** STR profiles of RT112 and RT4.


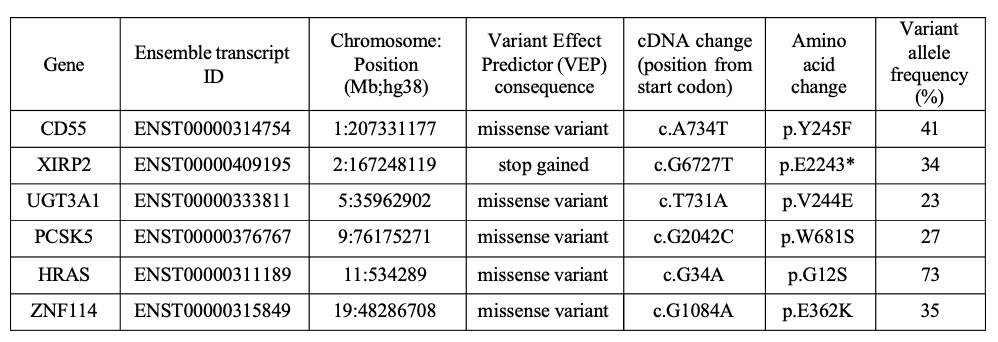


**Table S2.** Mutations identified in RT112 R3.


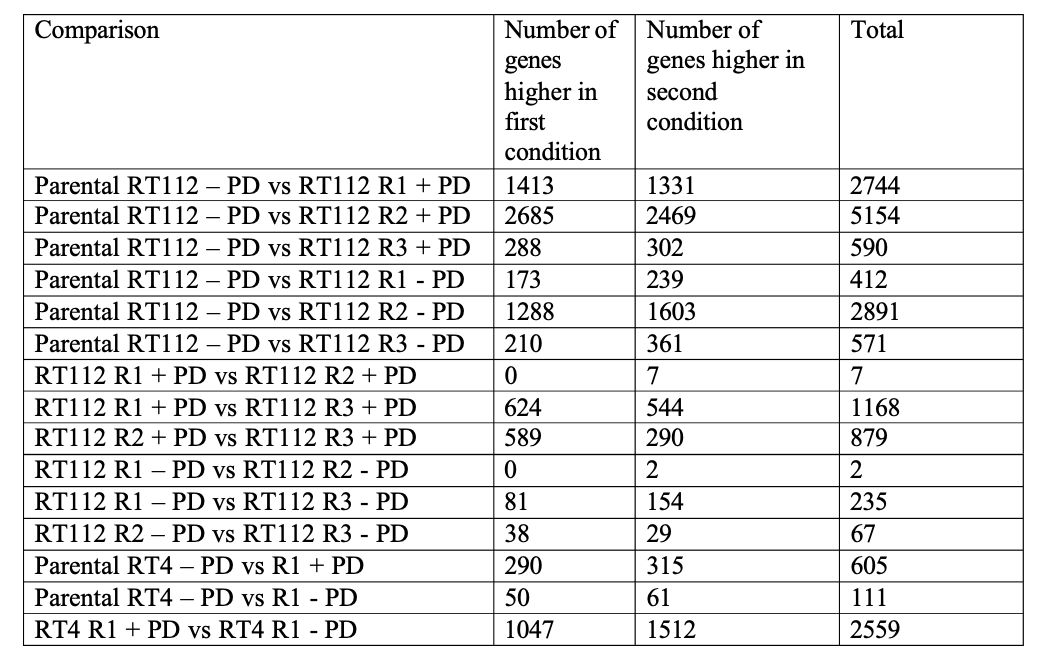


**Table S4.** Differentially expressed genes (limma test, FDR 0.01) in comparisons of RT112 and RT4 experimental conditions.
